# An MR fingerprinting approach for quantitative inhomogeneous magnetization transfer imaging

**DOI:** 10.1002/mrm.28984

**Published:** 2021-08-21

**Authors:** Daniel J. West, Gastao Cruz, Rui P. A. G. Teixeira, Torben Schneider, Jacques-Donald Tournier, Joseph V. Hajnal, Claudia Prieto, Shaihan J. Malik

**Affiliations:** 1 Department of Biomedical Engineering, School of Biomedical Engineering and Imaging Sciences, King’s College London, St. Thomas’ Hospital, London, United Kingdom; 2 Centre for the Developing Brain, School of Biomedical Engineering and Imaging Sciences, King’s College London, St. Thomas’ Hospital, London, United Kingdom; 3 Philips Healthcare, Guildford, United Kingdom

**Keywords:** dipolar order, ihMT, magnetization transfer, MR fingerprinting, myelin imaging

## Abstract

**Purpose:**

Magnetization transfer (MT) and inhomogeneous MT (ihMT) contrasts are used in MRI to provide information about macromolecular tissue content. In particular, MT is sensitive to macromolecules, and ihMT appears to be specific to myelinated tissue. This study proposes a technique to characterize MT and ihMT properties from a single acquisition, producing both semiquantitative contrast ratios and quantitative parameter maps.

**Theory and Methods:**

Building on previous work that uses multiband RF pulses to efficiently generate ihMT contrast, we propose a cyclic steady-state approach that cycles between multiband and single-band pulses to boost the achieved contrast. Resultant time-variable signals are reminiscent of an MR fingerprinting acquisition, except that the signal fluctuations are entirely mediated by MT effects. A dictionary-based low-rank inversion method is used to reconstruct the resulting images and to produce both semiquantitative MT ratio and ihMT ratio maps, as well as quantitative parameter estimates corresponding to an ihMT tissue model.

**Results:**

Phantom and in vivo brain data acquired at 1.5 Tesla demonstrate the expected contrast trends, with ihMT ratio maps showing contrast more specific to white matter, as has been reported by others. Quantitative estimation of semisolid fraction and dipolar T_1_ was also possible and yielded measurements consistent with literature values in the brain.

**Conclusion:**

By cycling between multiband and single-band pulses, an entirely MT-mediated *fingerprinting* method was demonstrated. This proof-of-concept approach can be used to generate semiquantitative maps and quantitatively estimate some macromolecular-specific tissue parameters.

## 1 Introduction

Magnetization transfer (MT) and inhomogeneous MT (ihMT) contrasts are used in MRI to provide information about macromolecular tissue content. In particular, MT is sensitive to macromolecules, and ihMT appears to be specific to substances with non-zero dipolar magnetization order, including the lipid bilayers that form myelin.^1–4^ Recently, ihMT measurements have compared favorably against other myelin imaging metrics,^5,6^ correlated strongly with fluorescence microscopy findings,^7^ and provided sensitivity for the assessment of demyelinating conditions such as multiple sclerosis.^8^ MT and ihMT contrasts are usually obtained by sequences with off-resonance RF saturation pulses (that only affect the semisolid pool), followed by readout periods for measurement. For example, ihMT gradient echo methods have been developed and optimized to achieve high-resolution whole-brain imaging at 1.5 Tesla.^9^

The ihMT effect arises because semisolid magnetization can be modelled as containing pools of both Zeeman and dipolar order that can be made to exchange by the presence of off-resonant RF irradiation; dual-frequency saturation with equal and opposite frequency offsets cancels this interaction. In our previous work, it was shown that ihMT contrast can also be generated using nonselective multiband pulses that perform off-resonance saturation and on-resonance excitation simultaneously.^10^ These pulses were originally proposed to control for MT effects in variable flip angle techniques by ensuring constant RF power across all flip angles, resulting in more stable relaxometry measurements.^11^ In order to generate ihMT contrast, images using 2-band excitation (1 on-resonance and 1 off-resonance band) leading to Zeeman-dipolar coupling were compared with 3-band excitation (1 on-resonance and 2 equal and opposite off-resonance bands) in which this coupling is canceled. Use of the same total power results in the same classical MT effect for tissues in which dipolar order can be neglected.

Resultant ihMT ratios (ihMTRs) from this type of sequence are generally small, for example ~4% in white matter (WM), which is similar to other steady-state measurement techniques.^9^ In order to boost this contrast, Varma et al. recently proposed use of a low duty-cycle RF saturation scheme in which saturation pulses are concentrated into short time periods with interleaved recovery time during which data are acquired.^12^ With this as motivation, in this work we propose a modulated cyclic steady-state sequence employing a balanced steady-state free precession (bSSFP) acquisition with multiband pulses that are alternated over time to create a similar low duty-cycle saturation effect. Each RF pulse contains an on-resonance component, meaning that data are continuously read out, improving the efficiency of encoding.

By holding the on-resonance flip angle constant while periodically alternating off-resonant saturation, the proposed sequence results in signal fluctuations that are entirely mediated by MT. This time-varying signal is then used for quantitative parameter estimation by applying current MR fingerprinting^13^ (MRF) methods. Images are spatially encoded using an undersampled tiny golden angle radial *fc*-space trajectory^14^ and reconstructed using dictionary-based low-rank inversion^15^ previously used for MRF. The proposed sequence is essentially an MRF acquisition but one that should yield only constant signals if tissues follow the standard Bloch equations. Because the induced signal variations are mediated by MT effects, we refer to our approach as *MT-MRF*.

Several studies have begun to quantify multicompartment tissue parameters using MRF. Kang et al.^16^ and Kim et al.^17^ estimate four parameters of a Bloch-McConnell system to synthesize MTC and CEST images, whereas Cohen et al.^18^ and Heo et al.^19^ quantify amide proton exchange rates and concentrations. The novelty of MT-MRF primarily comes from the estimation of dipolar parameter $T_{1\text{D}}^{s}$ using a sequence in which only off-resonant RF power is varied. Although not currently optimized for quantification, this work explores the encoding ability of MT-MRF in its basic form, as well as its potential as a quantitative ihMT method that can be compared to other approaches.^20^

In addition to generating quantitative parameter estimates, we also show that the reconstructed time-series data can be used to produce semiquantitative MTR and ihMTR maps. We present an evaluation of the proposed method using phantoms, as well as MT-MRF brain images acquired from 5 healthy subjects. A comparison with a previous steady-state method is also included.^10^

## 2 Theory

### 2.1 Sequence description

**Figure 1:**
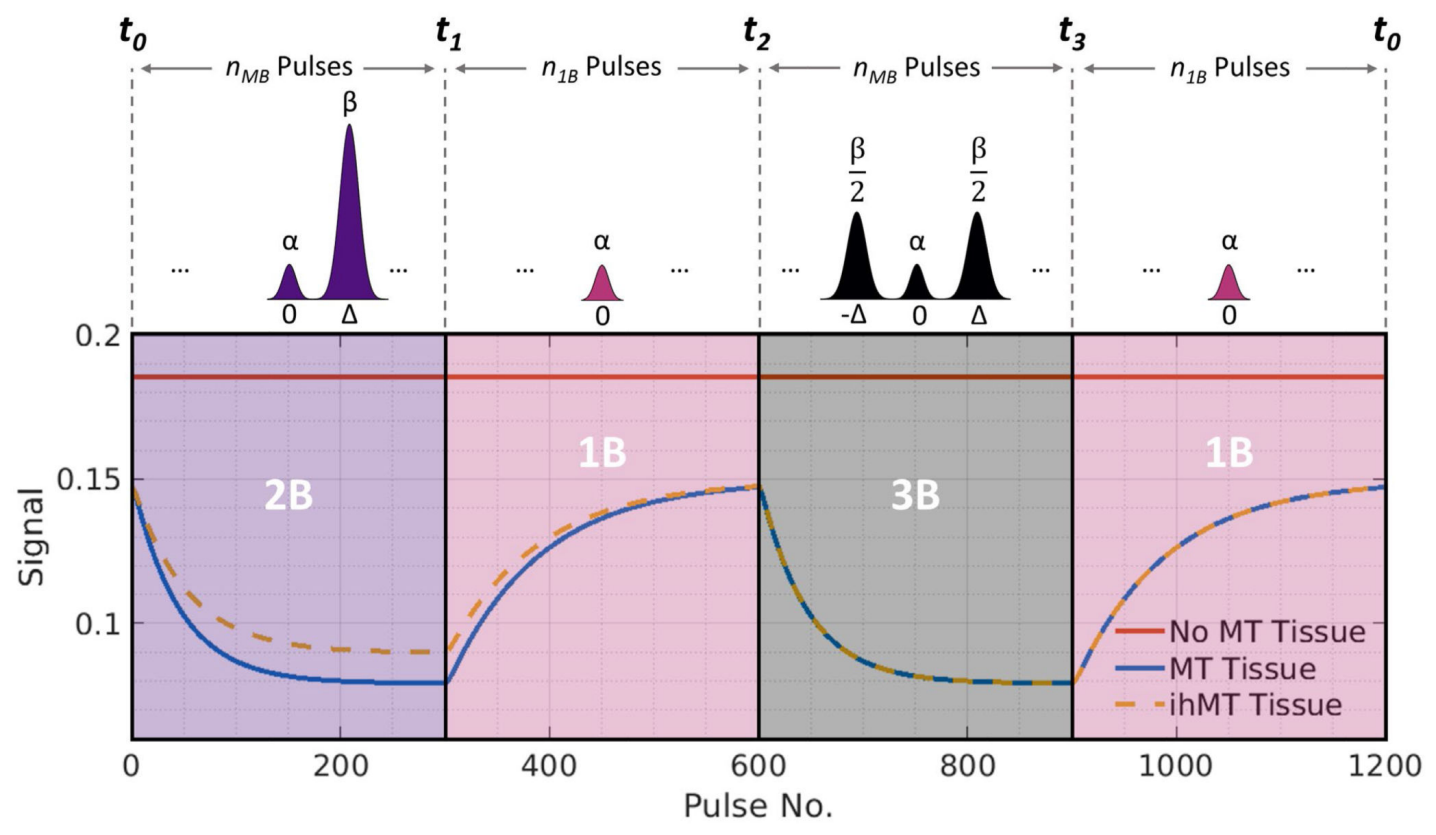
The proposed acquisition uses a rapid gradient echo sequence alternating between the pulses shown in the top row in blocks (bSSFP is used in this work). The on-resonance part of each pulse (with power α) is identical; thus, free water (no MT effect) would give constant signal throughout the acquisition. The switched off-resonance bands will affect semisolid proton saturation, resulting in modulation of the free water signal via MT as shown. 2-band (2B) and 3-band (3B) pulses are power-matched (with a combined off-resonance power β) and alternated to give ihMT contrast. Abbreviations: bSSFP, balanced steady-state free precession; ihMT, inhomogeneous magnetization transfer; MT, magnetization transfer; *n*_1B_, number of single-band pulses; *n*_MB_, number of multiband pulses

The proposed sequence consists of a 3D bSSFP acquisition with constant flip angle (FA) and TR, in which the excitation pulses are periodically switched between single-band and multiband pulses with different variants, as shown in Figure 1. One cycle comprises a number, *n*_MB_ of 2-band pulses, followed by a block of *n*_1B_ single-band pulses, a block of *n*_MB_ 3-band pulses, then another block of *n*_1B_ single-band pulses (to allow semisolid saturation to reduce before the next cycle). The on-resonance bands of each pulse are identical; hence, a tissue with no semisolid compartment would give constant signal. Tissues with a semisolid component respond transiently because they saturate strongly during multiband periods due to off-resonant bands and recover during single-band periods. Tissues with significant dipolar order effects will also respond differently to the 2-band and 3-band pulses. Example signal curves are shown in Figure 1.

The sequence repeats many times; the total number of excitations in one cycle (N_cycle_ = 2*n*_MB_ + 2*n*_1B_) is typically of the order of 1000 such that the cycle repeats after a few seconds for the TRs (~5 ms) achieved using bSSFP with multiband pulses. After a small number of repeats, the signal reaches a cyclic steady-state in which MT or ihMT contrast is dictated by parameters such as *n*_1B_ and *n*_MB_, as well as FA, TR, off-resonance power, and offset frequency. Qualitative comparison of the efficiency of ihMT contrast generation for different parameter combinations is achieved by defining *η* as peak ihMT contrast generated per square root of time, in which TR is the sequence repetition time. Times *t*_1_ and *t_3_* are at the ends of 2B and 3B pulse periods, as in Figure 1.

(1) $$\eta =\frac{S\left(t_{1}\right)-S\left(t_{3}\right)}{\sqrt{\text{TR}}}=\frac{\Delta \text{ihMT}}{\sqrt{\text{TR}}}$$

### 2.2 Spatial encoding and image reconstruction

The cycling of the acquisition sequence can repeat indefinitely and be made independent of spatial encoding. Because the pulses are spatially nonselective, the encoding must be 3D and cover the entire FOV (the head). Full 3D characterization of each of the N_cycle_ different contrast states is infeasible using regular encoding methods. Instead, we take advantage of the fact that changes in signal are compressible in the temporal direction and can be described by a low-rank representation determined from a dictionary of simulations computed for a range of expected tissue properties. As shown by McGivney et al.,^21^ the dictionary **D** can be compressed by singular value decomposition; in addition, its low-rank approximation can be obtained by multiplication of the dictionary and the matrix of left singular vectors **U**, truncated to a certain rank R to yield **U_R_** ϵ ℂ^TNC^×^RNC^, where T is the number of acquired timepoints; N is number of voxels; and C is number of coils. The reconstruction problem was previously formulated for MRF as^15,21,22^:

(2) $$\underset{\tilde{\text{x}},{\text{𝒯}}_{b}}{\text{argmin}∥}{\text{U}}_{\text{R}}\text{F}C\tilde{\text{x}}-\text{s}{∥}_{2}^{2}+\lambda \underset{b}{\sum ∥}{\text{𝒯}}_{b}{∥}_{*}s.t.{\text{𝒯}}_{b}={\text{P}}_{\text{b}}(\tilde{\text{x}}).$$

Here, **S**ϵℂ^TKC^ is the *k*-space signal where K is *k*-space trajectory length. In Equation (2), **F**ϵℂ^RNC×RNC^ performs nonuniform fast Fourier transform and gridding operations; **C**ϵℂ^RNC×RN^ represents coil sensitivity maps; **x** is a time-series of images sought to be reconstructed; and $\tilde{\text{x}}$ is its low-rank approximation (**x** = **U_R_**$\tilde{\text{x}}$, thus $\tilde{\text{x}}={\text{U}}_{\text{R}}^{\text{H}}\text{x}$, where **x**ϵℂC™ and $\tilde{\text{x}}\in {\mathbb{C}}^{\text{RN}}$). Patch-based regularization was added to the problem, as in Bustin et al.^23^ **P_b_** constructs a 3D local tensor *𝓙_b_* around voxel *b* by concatenating local (in each patch), nonlocal (between similar patches in a neighborhood), and contrast voxels along each dimension.^24,25^ This problem can be solved using the alternating direction method of multipliers^26^; further details can be found in Ref. [23].

There is some flexibility in how much data needs to be collected and how this should be spatially encoded. It has been found that radial or spiral *k*-space encoding leads to better conditioning of the reconstruction problem than Cartesian sampling.^27–29^ Furthermore, the framework allows for a flexible amount of undersampling to be used. In total, the amount of data collected would be sufficient to reconstruct N_V_ fully sampled volumes (ignoring temporal modulation of the signal), whereas the number of actually reconstructed volumes is R.

## 3 Methods

### 3.1 Signal model and efficient simulations

The tissue model used in this work^1^ consists of a single pool of free-water protons, denoted *f*, and a semisolid pool that contains two subcompartments: one without dipolar order effects, denoted *s*1 (fractional size 1 – *δ*); and the other with dipolar order, denoted *s*2 (fractional size *δ*). Key model parameters include: free proton T_1_ and T_2_, $T_{1}^{f}$ and $T_{2}^{f}$; semisolid Zeeman and dipolar ${\text{T}}_{1}\text{s}$, $T_{1Z}^{s}=T_{1Z}^{s1}=T_{1Z}^{s2}$ and $T_{1\text{D}}^{s}=T_{1\text{D}}^{s2}$ semisolid T_2_, $T_{2}^{s}=T_{2}^{s1}=T_{2}^{s2}$; fractional equilibrium magnetizations, $M_{0}^{f}$ and $M_{0}^{s}\left(M_{0}^{J}+M_{0}^{S}=1\right)$; $f=M_{0}^{s}/\left(M_{0}^{s}+M_{0}^{f}\right)$; exchange rate *k* between free and semisolid pools; and semisolid absorption lineshape $g\left(\text{Δ},T_{2}^{s}\right)$, defined for an offset frequency Δ.

Malik et al.^10^ proposed an efficient means for simulating the steady-state behavior of a sequence in which the evolution over multiple RF pulses and delay periods can be described by a single homogeneous eigenvalue expression. Specific details for MT-MRF are in Supporting Information Section 1.

### 3.2 Sequence design

**Figure 2:**
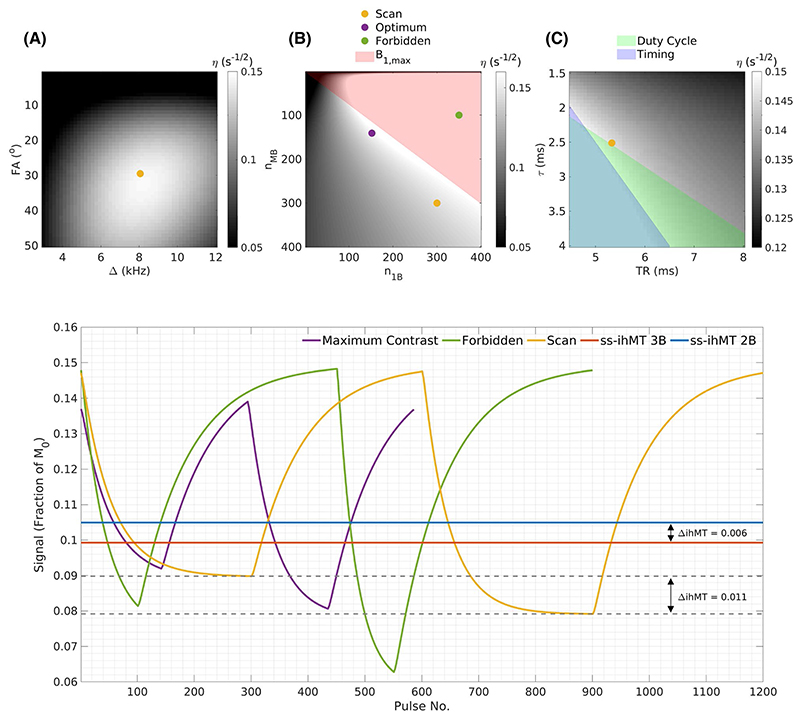
*Top: η* as a function of sequence parameters (FA, Δ, *n*_MB_, *n*_1B_, *τ*, TR). In each subfigure, non-plotted parameters are fixed to: TR = 5.3 ms, FA = 29.5°, *τ* = 2.5 ms, Δ = 8.1 kHz, B_1,rms_ = 4 μT, and *n*_MB_ = *n*_1B_ = 300. (A) Efficiency as a function of frequency offset and on-resonance FA. (B) Efficiency as a function of cycle duration parameters *n*_MB_ and *n*_1B_. The colored dots mark exemplar schemes plotted below. *Scan* parameters are those listed in this caption. (C) Efficiency as a function of TR and pulse duration, showing the operation of timing and duty-cycle constraints. Colored areas are inaccessible parameter combinations that violate hardware constraints. *Bottom:* Signal profiles simulated for the dotted schemes above, and ss-ihMT with equivalent B_1,rms_. The time-variable sequences are ordered 2B-1B-3B-1B (i.e., 2-band pulse period, then 1-band, etc). ihMT contrast is the difference in signals at the end of the 2B and 3B periods. *Scan* parameters generate greater contrast than the steady-state sequence. The *maximum contrast* sequence obtains slightly higher contrast, whereas the *forbidden* scheme (green) yields far higher contrast but violates peak B_1_ constraints. Abbreviations: FA, flip angle; ss-ihMT, steady-state inhomogeneous magnetization transfer

Sequences of the type shown in Figure 1 were simulated using internal capsule tissue parameters from Mchinda et al.^9^ and *η* values calculated for different parameter ranges, as shown in Figure 2. Several constraints were considered according to scanner hardware limitations: maximum pulse amplitude, B_1,max_ < 20 μT; TR > 2τ (due to RF amplifier duty-cycle limits); and a timing constraint, TR > 2.5 + *τ*, where 2.5 ms is readout duration and *τ* is RF pulse duration. Simulated signals were compared to our previous steady-state ihMT (ss-ihMT) method with scan parameters matching the in vivo scheme used in Malik et al.^10^ The *maximum contrast* solution in Figure 2 was found using a genetic algorithm (*ga* in MatLab 2019a; MathWorks, Natick, MA). The *scan* case was used for all experiments.

The sequence design for MT-MRF was chosen to give large ihMT contrast, with the constraint that all on-resonance flip angles be constant, that is, that all signal fluctuations are MT-mediated only.

**Figure 3:**
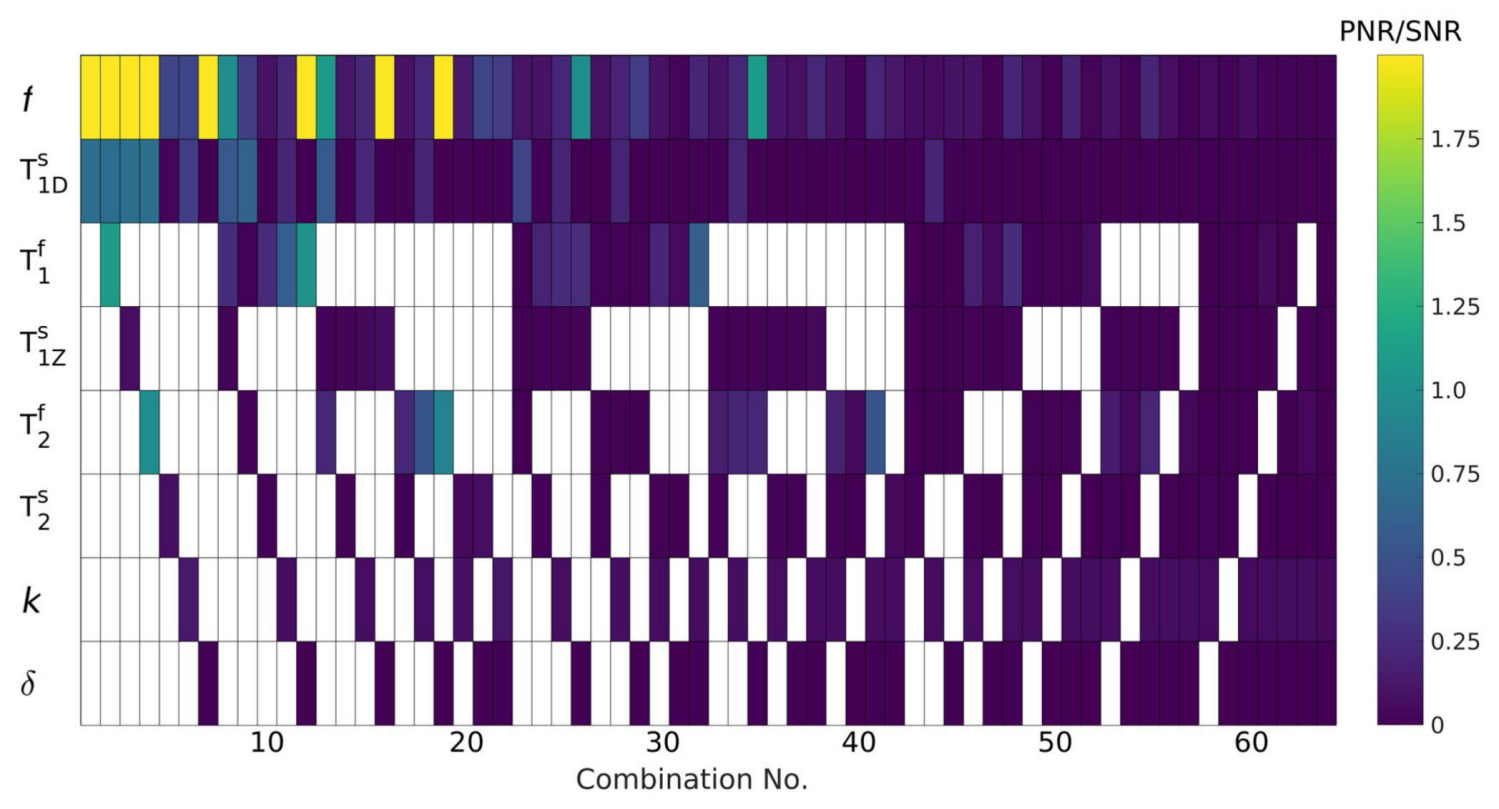
Calculation of PNR/SNR for different parameter combinations. Colored squares indicate parameters that are estimated for a given combination (column), whereas white squares indicate fixed parameters (i.e., the left-most columns feature the most fixed parameters, and the right-most columns feature the most estimated parameters). To achieve a reasonable PNR, only three parameters can be estimated simultaneously. Combination 2 is used for all dictionary fits. Abbreviations: PNR, parameter-to-noise ratio

To understand how well this sequence could be used to estimate underlying model parameters, we computed the Cramér-Rao lower bound (CRLB)^30–33^ and used this to estimate parameter-to-noise ratios. Because the parameter-to-noise ratio and image SNR are both dependent on the underlying acquisition noise level, we have quoted the ratio parameter-to-noise ratio/SNR that independently quantifies the precision relative to image SNR. Figure 3 shows parameter-to-noise ratio/SNR when estimating different combinations of parameters with others fixed at their true values (fixed parameters shown as white). For all further analysis, WM/gray matter (GM)/GM-WM average parameter sets from Varma et al.^12^ are considered and correspond to respective values: $R_{1}^{f}$ (i.e., $1/T_{1}^{f}$) = 0.92/0.55/0.735 s^–1^, $T_{1Z}^{s}$ = 1/1/1 s, $T_{2}^{f}$ = 69/99/84 ms, $T_{1\text{D}}^{s}$ = 6.2/5.9/6.05 ms, $T_{2}^{s}$ = 9/7.58/8.28 μs, *k* = 59.6/50.8/55.2 s^–1^, and *f* = 0.101/0.0348/0.0679. *δ* = 1 (i.e., a single semisolid compartment model) is used to simplify parameter estimation as per recent quantitative ihMT studies.^10,12^ From Figure 3, it appears that $T_{1}^{f}$, *f*, and $T_{1D}^{s}$ can be estimated to reasonable precision with others held fixed (combination 2), which motivates the approach taken in subsequent sections.

### 3.3 Dictionary generation, low-rank basis, and parameter estimation

Two types of dictionaries were required for this work: a *full dictionary* **D** and *reduced dictionary* $\tilde{\text{D}}$. First, **D** included variation of all model parameters. Having 9 free parameters and a long temporal duration of each atom (1200 readouts) meant that the dictionary would quickly become too large for the memory of the PC used for calculation (8(16) × Intel Core i7-5960X 3.00 GHz CPU, 64 GB RAM; Intel Corporation, Santa Clara, CA). Hence, a version of **D** was constructed using coarse sampling with the sole purpose being to discover a lower rank basis, as detailed above. Second, $\tilde{\text{D}}$ was constructed for parameter estimation using finer sampling of the three estimated parameters (with all others fixed).

**D** was created with ~1 million atoms using parameter value ranges: $T_{1}^{f}$ = 0.2:0.5:3.7 s, $T_{1\text{Z}}^{s}$ = 0.1:0.2:0.9 s, $T_{1\text{D}}^{S}$= 2:4:22 ms, $T_{2}^{f}$ = 50:70:470 ms, $T_{2}^{S}$ = 5:3:20 μs, *δ* = 0:0.2:1, *k* = 40:20:100 s^–1^, *f* = 0:0.05:0.25, and B_0_-induced phase per TR = –π:π/3:π because the method is based on bSSFP. The semisolid lineshape was assumed to be super-Lorentzian.^34^

**Figure 4:**
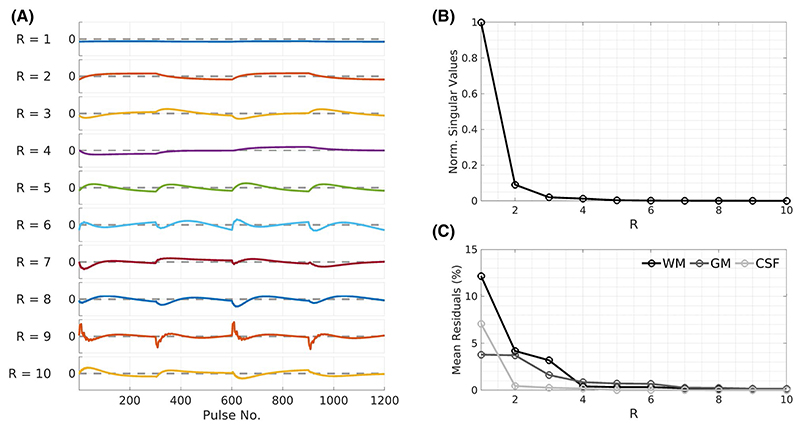
Low-rank analysis of the dictionary **D**. (A) The first 10 singular vectors of the dictionary; each axis is scaled identically to a [–0.2-0.2] range. Each subplot represents one column of **U**_R_, and dashed lines indicate the position of 0 along each *y*-axis. (B) Singular values corresponding to each singular vector, normalized to the first singular value. (C) Mean residuals between simulated WM, GM, and CSF signal curves and equivalent low-rank approximations for different R. The three plausible MT-MRF signals are better represented as R increases and are sufficiently described by R = 5. Abbreviations: CSF, cerebrospinal fluid; GM, gray matter; MT-MRF, magnetization transfer-mediated MR fingerprinting; WM, white matter

The low-rank basis **U**_R_ was constructed by performing a singular value decomposition on **D**. A further reduction by randomly sampling 600,000 atoms was needed to perform the singular value decomposition; the random sampling was done ensuring an equal number of “no semisolid” (*f* = 0), “no dipolar order” (*f* ≠ 0, *δ* = 0), and “ihMT” entries (*f* ≠ 0, *δ* ≠ 0). The ability of the resulting basis to reproduce arbitrary tissue signals was assessed by projecting some test signals into the low-rank space via multiplication by ${\text{U}}_{\text{R}}^{\text{H}}$ (truncated to different ranks) and then back into the time domain by **U**_R_. These approximated signals were compared to their groundtruth simulated equivalents (Figure 4). Test signals were not themselves dictionary atoms but represented expected tissue values for cerebrospinal fluid (CSF), GM, and WM. CSF was assigned $T_{1}^{f}$ = 3 s, $T_{2}^{f}$ = 2 s, and *f* = 0, whereas GM/WM took their respective values from Section 3.2.

Reduced dictionary $\tilde{\text{D}}$ was generated to estimate only $T_{1}^{f}$, *f*, and $T_{1\text{D}}^{s}$ simultaneously using 100 increments of each parameter over their feasible ranges. The size of this dictionary was further reduced using the low-rank basis discovered from **D** such that more increments could have been used if required. It was found that exceeding 100 increments for each parameter was unnecessary, with changes below the noise level when trialed on the acquired data. Unless stated otherwise, all reduced dictionaries used $T_{1Z}^{S}$ = 1 s, $T_{2}^{f}$ = 84 ms, $T_{2}^{S}$ = 8.28 μs, and *k* = 55.2 s^–1^ (GM-WM averages). To assess estimation bias, a numerical simulation was performed in which MT-MRF signals were forward simulated using different $T_{1Z}^{S}$, $T_{2}^{f}$, $T_{2}^{S}$ and *k* values before estimating $T_{1}^{f}$, *f*, and $T_{1\text{D}}^{S}$ using identical $\tilde{\text{D}}$ (i.e., without changing fixed parameters). Further details are in Supporting Information Section 6.

Reconstructed data ($\tilde{\text{x}}$) can be projected back to the time domain (**X**) from which familiar semiquantitative metrics, MTR and ihMTR, can be calculated. The degree of contrast varies through time (because the signals are time-varying); thus, we define these metrics using the minimum and maximum signal values, where *t*_0_ to *t*_3_ are times within the cycle (see Figure 1):

(3) $$\text{MTR}=1-\frac{S\left(t_{1}\right)+S\left(t_{3}\right)}{S\left(t_{0}\right)+S\left(t_{2}\right)}$$

(4) $$\text{ihMTR}=2\frac{S\left(t_{1}\right)-S\left(t_{3}\right)}{S\left(t_{0}\right)+S\left(t_{2}\right)}$$

### 3.4 Imaging experiments

All experiments were performed on a 1.5 Tesla Philips Ingenia MRI system (Philips, Best, Netherlands) with a 15-channel head coil for signal reception.

#### 3.4.1 System characterization and correction for RF instability

Our sequence was first characterized using 1D phantom scans (test-tube phantom with no phase encoding), allowing direct measurement of the full temporal signal with no need for low-rank reconstruction. Inconsistencies were found between the on-resonance amplitudes and phases of each RF pulse type, causing oscillations in signal when switching between them. The origin of these inconsistencies could not be determined but were stable for a given pulse configuration. Empirically determined complex scaling factors for each pulse were used to ensure the on-resonance component of each pulse type was the same. See Supporting Information Section 2 for more details.

#### 3.4.2 Sequence implementations

For MT-MRF, a 3D tiny golden angle radial, “stack-of-stars” *k*-space trajectory with one spoke per RF pulse was used for all experiments,^14^ where TR = 5.3 ms, TE = 2.7 ms, and FA = 29.5° (additional sequence details below). Encoding was ordered to loop over repeats of each radial “star” before then looping over *k_z_* partitions. Although not necessary for the method to work, the total number of radial spokes acquired per partition was set to be commensurate with N_cycle_ such that the same *k_x_*–*k_y_* sampling was used for all *k_z_* partitions, enabling independent reconstruction of each *z*-location. As a reference, ss-ihMT^10^ was also used, in this case with Cartesian *k*-space sampling, acquiring separate images using 1-band, 2-band, and 3-band RF pulses.

#### 3.4.3 Phantom validation

MT-MRF data were acquired on three phantoms: MnCl_2_-doped water (0.05 mM), cross-linked bovine serum albumin (10% w/w in water prepared as in Koenig et al.^35^), and prolipid 161 (PL161) (15% w/w; Ashland Inc, Covington, KY; prepared as in Swanson et al.^2^ but excluding any T_1_ reducing agent). These phantoms are examples of “no semisolid”, “no dipolar order”, and “ihMT” materials, respectively. FOV of 115 × 115 × 150 mm and isotropic resolution 1.5 mm^3^ were used with a total acquisition time of 21 minutes 32 seconds. Quantitative maps of $T_{1}^{f}$, *f*, and $T_{1\text{D}}^{s}$ were computed using the reduced dictionary. In practice, it was observed that the large spread of $T_{2}^{f}$ and $T_{1}^{f}$ between phantoms led to inconsistent results; thus, a second fit was performed using an alternative dictionary with the same fixed parameters except $T_{2}^{f}$ = 130 ms. Quantitative parameter estimates were compared against ss-ihMT results from the same phantoms, acquired as part of a previous study.^10^ That study used the same resolution but acquired data over multiple excitation flip angles with a significantly longer total scan duration of 60 minutes 48 seconds. Quantitative maps were produced by dictionary-matching using separate dictionaries relevant to this sequence.

#### 3.4.4 In vivo study

Human scanning was performed on 5 healthy subjects (3 males and 2 females, age 29 ± 7.5 years) who gave prior written consent in line with local ethical approval. MT-MRF experiments used FOV = 250 × 250 × 180 mm and 1.5 mm^3^ isotropic resolution with sagittal orientation to avoid fold-over artefacts. 2400 spokes (2 × N_cycle_) were acquired per slice encode position, equating to N_V_~9 and a scan time of 25 minutes 48 seconds. One subject was scanned twice using MT-MRF (8 months apart). Quantitative maps of $T_{1}^{f}$, *f*, and $T_{1\text{D}}^{S}$ were computed using $\tilde{\text{D}}$. To corroborate numerical phantom results in Supporting Information Figures S6A-S6D, in vivo fits were repeated by changing fixed parameter values by ±10% (see Supporting Information Figure S6E).

For comparison, ss-ihMT datasets were also acquired for every subject. These acquisitions used the same resolution, FOV, TR, TE, and FA as MT-MRF scans; 6 signal averages were used to match acquisition duration (each average = 1 minute 23 seconds). For ss-ihMT data, motion correction using rigid body registration (*imregister* in MatLab 2019a; MathWorks) was run *before* signal averaging and subsequent calculation of MTR/ihMTR as in Ref. [10]. Spatial smoothing with Gaussian kernels (SD 1.05 mm for MTR and 1.35 mm for ihMTR) was used to improve SNR. Datasets for each subject were aligned. Regions-of-interest were drawn in corticospinal tracts, frontal WM, and cortical GM for numerical analysis. ss-ihMT can provide MTR/ihMTR but no other parameter estimates.

## 4 Results

### 4.1 Simulation of obtainable contrast

Figure 2 shows ihMT contrast efficiency (*η*) calculated for a range of acquisition parameters. The colored areas are excluded because they violate constraints on peak B_1_, duty-cycle, or timing. A clear region of maximum *η* exists between FA = 25°-40° and Δ = 7-9 kHz. Given the optimal FA and Δ, Figure 2B shows some example sequences with different *n*_1B_ and *n*_MB_ in a single cycle, marked by colored dots. The purple scheme has maximal contrast, although the dot marked scan (yellow) was used for our experiments, with sequence parameters: TR = 5.3 ms, FA = 29.5°, *τ* = 2.5 ms, Δ = 8.1 kHz, *n*_MB_ = *n*_1B_ = 300, and a constant B_1,rms_ = 4 μT (maximum achievable). This scheme was selected because it yielded more gradual contrast variations than the maximum contrast case and is expected to provide favorable properties for reconstruction.^29,36^ A forbidden scheme was also simulated (green) that exceeds maximum B_1_ constraints but generates even greater ihMT contrast (ΔihMT).

Simulated signal profiles are also compared in Figure 2 according to the protocols highlighted in the above heat maps, and with an ss-ihMT scan with equal time-averaged RF power (B_1_,_rms_). One cycle of each sequence is shown using ordering 2B-1B-3B-1B. ΔihMT is found by comparing the end of the 2B period with the end of the 3B period (*t*_1_ and *t*_3_, as labeled in Figure 1). The scan sequence generates almost twice the contrast achieved using ss-ihMT. The respective efficiencies of contrast generation are *η* = 0.080 s^–1^ and *η* = 0.146 s^–1^. The maximum contrast sequence yields only a marginally (~6%) greater ihMT effect, although the forbidden scheme contrast is significantly higher.

### 4.2 Dictionary analysis

Figure 4 displays the singular vectors (Figure 4A) as well as corresponding singular values (Figure 4B) of a dictionary corresponding to the scan sequence. The first 5 components describe 99.2% of the “energy” within the dictionary. Figure 4C plots the mean residuals in representing WM, GM, and CSF “test” signals for different R. Improved representation of each tissue type occurs for higher R as expected but by R = 5, all three tissue types are accurately recreated with negligible residuals.

### 4.3 Image reconstructions and semiquantitative maps

**Figure 5:**
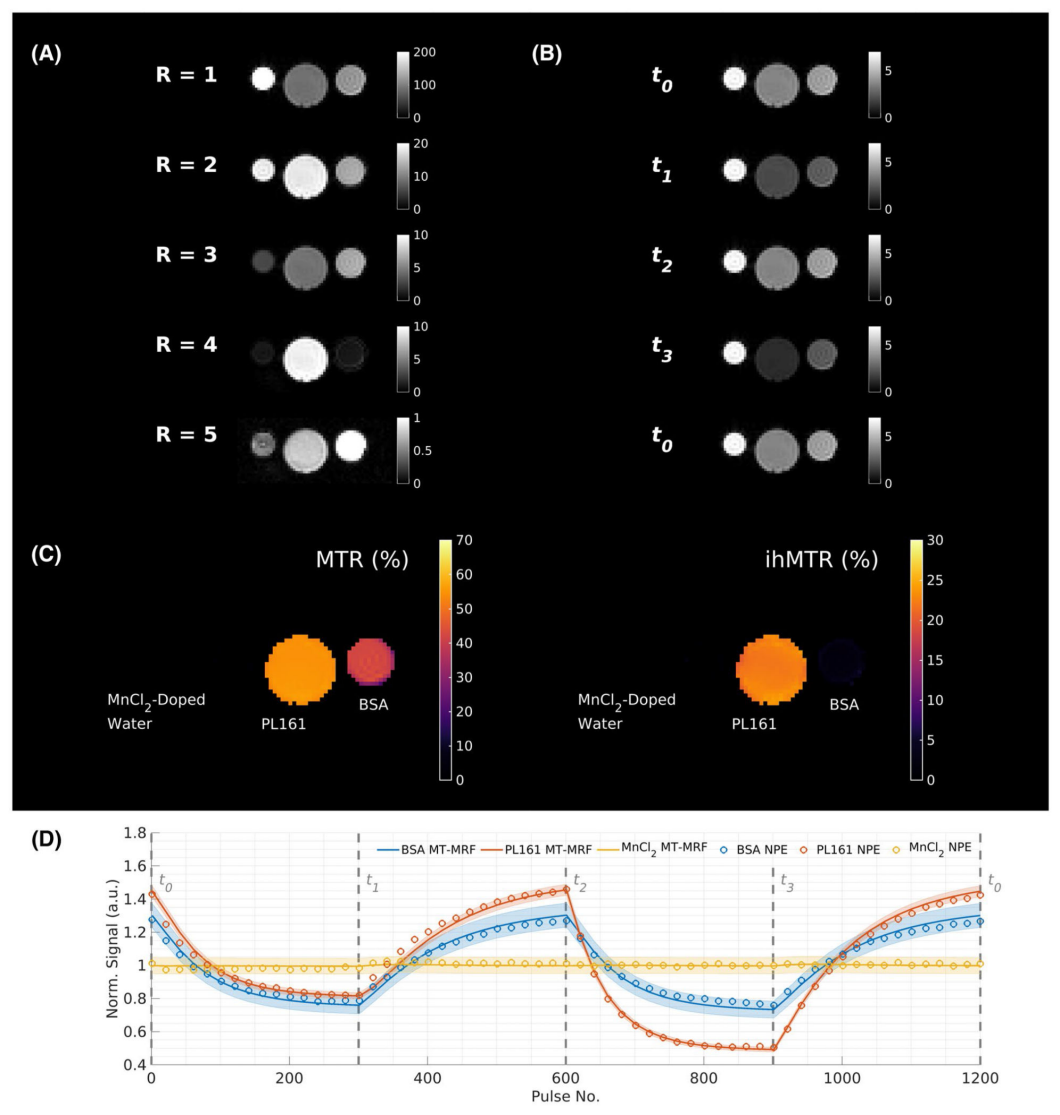
(A) Example single-slice images from the low-rank reconstructed phantom dataset ($\tilde{\text{x}}$). Tube locations are as labeled in (C). (B) The 5 displayed components are then transformed into the time domain to yield 1200 volumes of data; one slice is shown for the key timepoints labeled in Figure 1 (**x**). (C) Corresponding MTR and ihMTR maps show the expected contrast in each tube: ihMT effects are only seen in PL161 and MT effects, only in BSA and PL161; MnCl_2_-doped water has MTR = ihMTR = 0. (D) Signal time-courses from central regions-of-interest in each phantom compared to results from an NPE; the signal is faithfully represented by the low-rank reconstruction (these data are not fitted to one another, only overplotted). Abbreviations: BSA, bovine serum albumin; ihMTR, inhomogeneous magnetization transfer ratio; NPE, non-phase-encoded experiment; PL161, prolipid 161

Figure 5A displays a single slice from the MT-MRF phantom acquisition in the reduced basis (i.e., $\tilde{\text{x}}$); note the changes in intensity scale as R increases. Figure 5B shows example data projected back into the time domain, with MTR and ihMTR maps computed from these in Figure 5C. The MTR map shows non-zero values in bovine serum albumin and PL161 and no effect in MnCl_2_-doped water, as expected. The ihMTR map shows a significant effect only in PL161, again as expected. Region-of-interest measurements in the reconstructed images agree with direct non–phase-encoded (1D) measurements in Figure 5D, suggesting that the low-rank inversion reconstruction faithfully represents the time domain signal.

**Figure 6:**
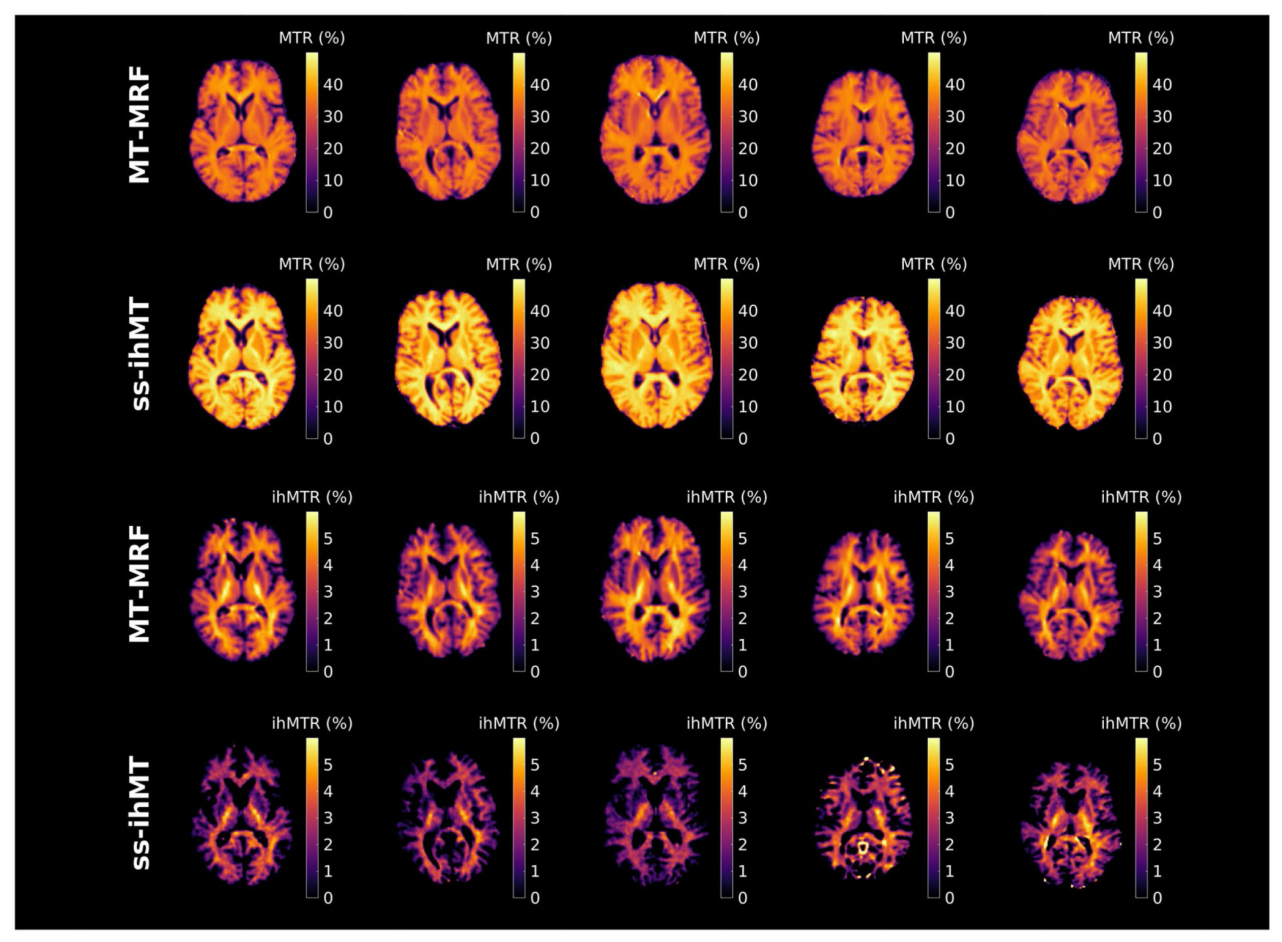
Example single-slice in vivo MTR and ihMTR maps from ss-ihMT and MT-MRF and each subject (separate columns). The former shows strong GM–WM contrast, but the latter consistently appears more correlated with WM. Abbreviations: MTR, magnetization transfer ratio

Figure 6 shows MTR and ihMTR maps of the brain from all 5 subjects. Table 1 and Supporting Information Table S2 compare these metrics between each method for matched regions-of-interest after image registration. Supporting Information Figure S4 is a test–retest comparison for the same subject scanned 8 months apart, and consistent contrast is observed. MT-MRF generally finds greater ihMTR than for ss-ihMT (62% greater in frontal WM, for example) but lower MTR; because the scheme was optimized for ihMT, the signal never fully returns to equilibrium. SDs in Table 1 are smaller for MT-MRF. This may be due to a better contrast-to-noise ratio, superior motion robustness because a radial readout was used, or use of spatial regularization (see Equation 2).

### 4.4 Quantitative parameter estimation

**Figure 7:**
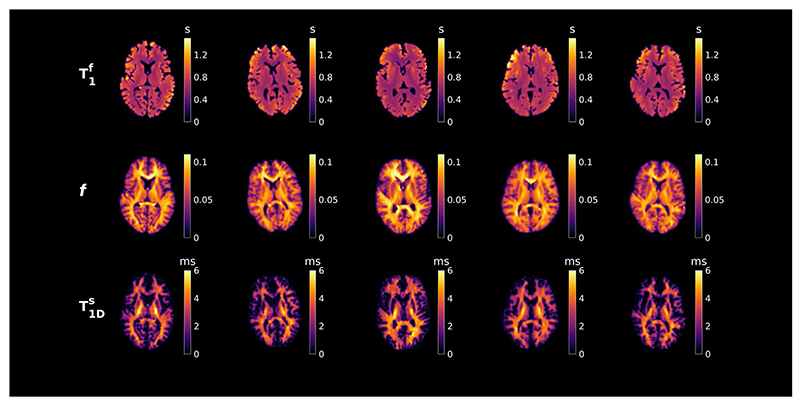
Estimation of free pool $T_{1}^{f}$, semisolid fraction (*J*), and dipolar $T_{1\text{D}}^{\text{s}}$ from a dictionary fit to in vivo data from each subject (separate columns). Maps for *f* show strong GM–WM contrast, but CST are more discernible in $T_{1\text{D}}^{s}$ maps. Abbreviations: CST, corticospinal tract

**Figure 8:**
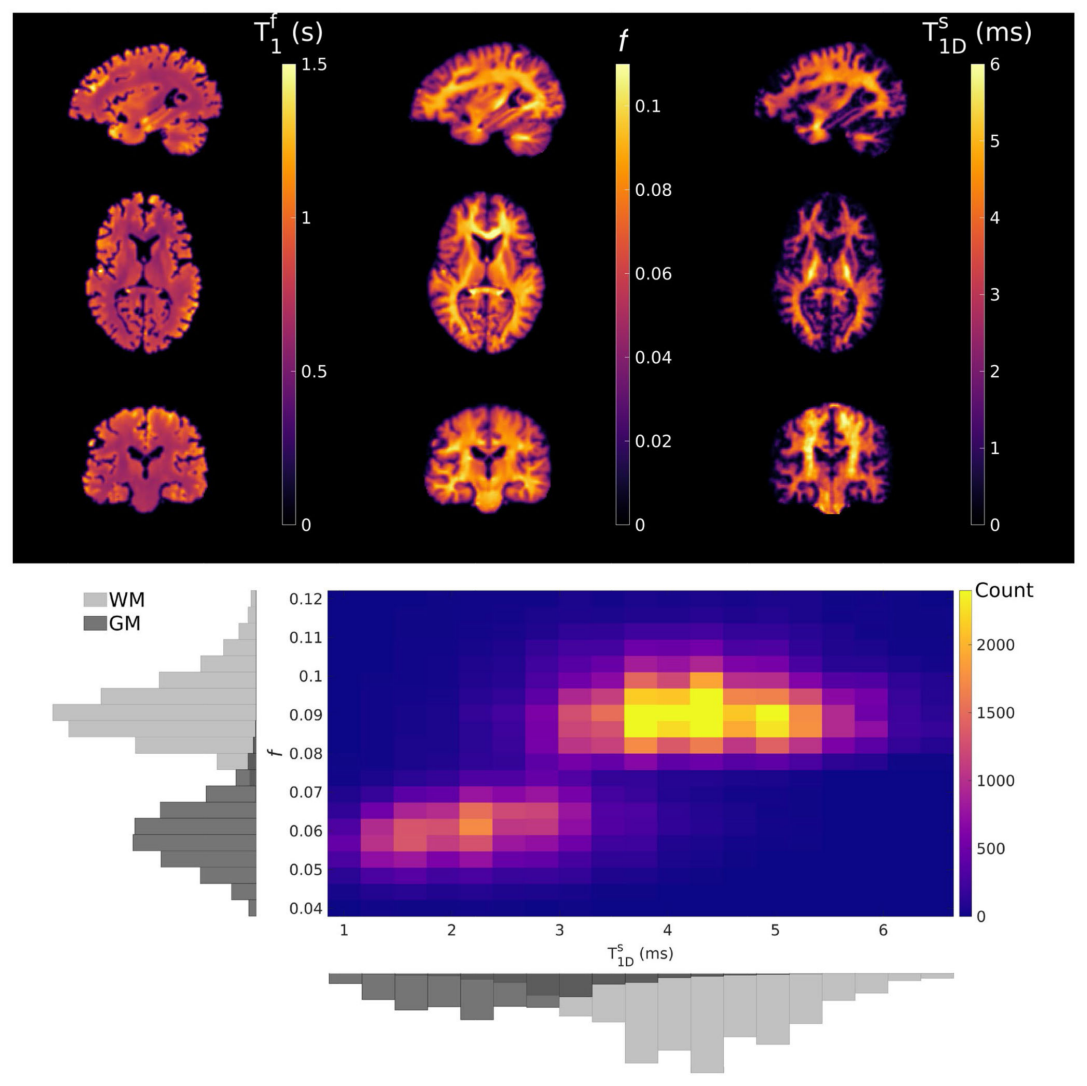
*Top*: Example parameter maps obtained from a dictionary fit to in vivo data from subject 1 (male, aged 41) in three orientations. *Bottom:* Joint-histograms of *f* and $T_{1D}^{s}$ produced from 3D GM and WM masks. These two parameters show different contrasts, and although strongly correlated, their relationship is not directly proportional, suggesting complementary information is provided

Figures 7 and 8 show example parameter maps for semisolid fraction *f*, dipolar relaxation time $T_{1\text{D}}^{S}$, and $T_{1}^{f}$. The phantom study (Table 2) compares values from MT-MRF against fitting to ss-ihMT data using multiple flip angles (details in Ref. [10]). Good agreement is observed between *f* and $T_{1D}^{s}$ estimates, with MT-MRF yielding tighter distributions (lower variance), although the ss-ihMT data were acquired over almost three times longer examination. Two sets of fixed parameters were used to estimate phantom properties ($T_{2}^{f}$ = 84 ms and $T_{2}^{f}$ = 130 ms; all others equal). Estimates of *f* and $T_{1\text{D}}^{s}$ are less sensitive to this change and agree within precision when using either fixed parameter set. $T_{1}^{f}$ changes significantly for bovine serum albumin and PL161 when fixed $T_{2}^{f}$ is altered. Corresponding MT-MRF parameter maps for these two scenarios are shown in Supporting Information Figure S3.

In vivo parameter maps from subject 1 are shown in Figure 8 for single axial, coronal, and sagittal slices. Figure 7 presents consistent parameter maps across all subjects, with *f* and $T_{1\text{D}}^{s}$ showing apparently distinct contrasts. To demonstrate this, a joint histogram of *f* and $T_{1\text{D}}^{s}$ in GM and WM pixels of subject 1 (masks created using FSL5.0 brain extraction tool and FMRIB's automated segmentation tool^37^) is included in Figure 8.

Both semiquantitative metrics (Figure 6, Table 1, and Supporting Information Table S2) and quantitative metrics (Figure 7, Table 1) show intersubject and test–retest (Supporting Information Figure S4) repeatability, with MT-MRF yielding a higher ihMTR across all chosen brain regions. Because tissues with *f* ~ 0 provide no MT-mediated contrast, $T_{1}^{f}$ cannot be estimated for these; this is the case for CSF and MnCl_2_-doped water. Consequently, $T_{1}^{f}$ map voxels with *f* < 0.04 are set to 0 because all GM and WM pixels in Figure 8 exceed this value.

## 5 Discussion

MT-MRF is essentially a very simple fingerprinting acquisition that would yield constant signals (and hence be unable to perform any parameter estimation) in the absence of MT. The resulting measurements are used for quantitative estimation of some of the underlying model parameters via dictionary-matching but also to reconstruct semiquantitative ratio maps.

### 5.1 MT-MRF for semiquantitative mapping

Although reconstruction of artefact-free time domain images is not usually associated with classic MRF methods, the low-rank inversion^15^ approach does produce high-quality “singular” images ($\tilde{\text{x}}$) that would normally be passed directly to dictionary-matching. It is also possible to map these back to the time domain (**x**), and we have used this property to produce MTR/ihMTR maps using points of maximum contrast within the time-evolving signals as references (Equations 3 and 4).

The initial motivation for developing MT-MRF was to improve the contrast achieved from the previously developed ss-ihMT method.^10^ Whereas ss-ihMT used separate image acquisitions with and without off-resonant saturation, it was shown by Varma et al.^12^ that a “low duty-cycle” scheme, alternating between high and low saturation power, would boost contrast. Sequence parameters were optimized with this in mind, trying to maximize the peak ihMT contrast (normalized to $\sqrt{\text{TR}}$). The extent of this improvement is shown in Figure 2; ihMT contrast is almost doubled versus ss-ihMT.

### 5.2 Quantitative parameter estimation

The generation of MTR and ihMTR maps is a limited use of the time-series data available; quantitative parameter estimation is also possible. CRLB calculation (Figure 3) suggested that not all parameters can be estimated to good precision; thus, it was decided to only reconstruct $T_{1}^{f}$, *f*, and $T_{1\text{D}}^{s}$. Phantom experiments using MT-MRF were compared to previous measurements using ss-ihMT with variable flip angles for estimation of these three parameters (Table 2). Estimates of *f* and $T_{1\text{D}}^{S}$ agree for both methods, and the MT-MRF sequence provides better precision, particularly for $T_{1\text{D}}^{s}$. The estimated ~ 25 ms for PL161 is in line with measurements from Swanson et al. Fits using different $T_{2}^{f}$ were performed because bovine serum albumin and PL161 have very different properties (more so than brain tissue); thus, fixing parameters was found to cause different $T_{1}^{f}$ estimation bias for MT-MRF and ss-ihMT. However, MT parameters estimates (i.e., *f* and $T_{1\text{D}}^{s}$) were unchanged regardless of $T_{2}^{f}$.

In vivo results in Figure 7 show consistent parameter values across subjects. *f* and $T_{1\text{D}}^{s}$ are significantly different in GM compared to WM, and $T_{1\text{D}}^{s}$ seems to highlight strongly myelinated structures (e.g., corticospinal tract). Generally, parameter estimates between different subjects are in agreement (Table 1), with the highest $T_{1\text{D}}^{s}$ values found in corticospinal tract (~5.5 ms) and lower values (~2.2 ms) found in cortical GM; orientation with respect to B_0_ may influence these values.^38^ Estimates for *f* exhibit higher precision, an observation that is supported by the CRLB analysis in Figure 3. Maps of *f* and $T_{1\text{D}}^{s}$ do show distinct contrasts. Figure 8 presents a whole-brain joint-histogram from a single subject and demonstrates that these parameters are correlated but not simply linearly dependent.

Compared to the literature, corticospinal tract $T_{1\text{D}}^{s}$ estimates (~6.0 ms in the coronal slice in Figure 8) are generally in agreement with those reported elsewhere,^20^ and WM estimates for *f* concur with those in Varma et al.^12^ GM *f* values may be slightly higher here compared to other studies due to a fixed exchange rate; 25% to 40% differences between GM and WM *k* values have been reported in the literature.^39,40^ Others^20,41^ have reported GM $T_{1\text{D}}^{s}$ to be similar to WM (~5.6 ms); however, Swanson et al. also found far shorter $T_{1\text{D}}^{s}$ in GM compared to WM from bovine spinal cord.^2^ The use of a fixed dipolar fraction in this work may also contribute to an enhanced $T_{1\text{D}}^{s}$ GM-WM contrast. Future iterations of this method will aim to quantify *δ* and $T_{1\text{D}}^{s}$ simultaneously.

Here we estimate three parameters given an acquisition time of ~25 minutes for a 3D volume. Quantitative estimation is not possible in vivo using ss-ihMT with comparable acquisition time. The method of Varma et al.^20^ only quantifies $T_{1\text{D}}^{s}$ and requires ~96 seconds for a single slice (TR = 2 seconds, 12 repetitions, and 4 acquisitions). For comparison with our work, assuming slices cannot be interleaved, acquisition of a 150 mm FOV using 25 slices (6 mm slice thickness) would take ~40 minutes.

### 5.3 Parameter estimation biases

Parameter fixing is common in quantitative MT imaging because model parameters can influence the signals in similar ways; thus, estimation is not well posed and may create bias. $T_{1}^{f}$, *f*, and $T_{1\text{D}}^{s}$ are estimated here, whereas $T_{1Z}^{s}$, $T_{2}^{f}$, $T_{2}^{s}$, and *k* are fixed. Similar methods have been used by others: Varma et al. fixed *k*, $\frac{kf}{T_{1}^{f}}$, $\frac{1}{T_{1}^{f}T_{2}^{f}}$, and $T_{2}^{s}$ to estimate $T_{1\text{D}}^{s}$; Hilbert et al.^42^ fixed $T_{1}^{f}=T_{1Z}^{s}$, *k*, and $T_{2}^{S}$ to estimate $T_{1}^{f}$,$T_{2}^{f}$, and *f* using MRF; and Cohen et al.^18^ fixed $T_{1}^{f}$,$T_{1Z}^{s}$, and $T_{2}^{s}$ in a CEST-MRF study.

In our work, *f* and $T_{1\text{D}}^{s}$ estimates are comparable to those found by others. The phantom experiment provided similar estimates from two different experiments, although separate fixed values of $T_{2}^{f}$ were considered. The main impact of changing $T_{2}^{f}$ is to alter the estimate of $T_{1}^{f}$, which could indicate that this is acting as a “nuisance” parameter to absorb uncertainties in the relaxation times; this is also seen in Supporting Information Figure S6C. Because tissue parameters are unlikely to remain constant under all pathophysiological conditions, Supporting Information Figures S6A-E explore estimation bias in numerical phantoms and in vivo, respectively, with these results showing good agreement. $T_{1Z}^{s}$ has negligible impact on all parameter estimates; however, altering *k* causes small changes in *f* and $T_{1\text{D}}^{S}$. Despite $T_{2}^{f}$ strongly influencing $T_{1}^{f}$ estimation (as mentioned above), it has little effect on *f* and $T_{1\text{D}}^{s}$. $T_{1\text{D}}^{s}$ and $T_{2}^{s}$ are most correlated, as has also been reported by Varma et al.^20^

Because a bSSFP readout was used throughout, B_0_ variation was accounted for in the generation of **D** but was set to 0 for $\tilde{D}$. B_1_ inhomogeneities were assumed to be negligible because all experiments were performed at 1.5 Tesla. Subsequent implementations of MT-MRF at higher field strengths may require acquisition of field inhomogeneity maps.

Recent work from Wang et al.^43^ has questioned the commonly made assumptions that $T_{1Z}^{s}$ can either be fixed at some simple value (i.e., 1 second) or set equal to $T_{1}^{f}$.^44,45^ Instead, Wang et al. suggest that $T_{1Z}^{s}$ is a major determinant of observed T_1_ and is strongly frequency-dependent; a $T_{1\text{Z}}^{s}$ of approximately 120 ms in WM should be expected at 1.5 Tesla. Figure 8 used the assumption that $T_{1Z}^{s}$ = 1 s, but we repeated in vivo dictionary fits for subject 1 using a fixed value of 200 ms. The result in Supporting Information Figure S5A shows substantially longer $T_{1}^{f}$, although largely unchanged *f* and $T_{1\text{D}}^{s}$. CRLB analysis (Figure 3) showed that $T_{2}^{f}$ could be estimated instead of $T_{1}^{f}$. This was also performed (fixing $T_{1}^{f}$ = 1.36 s from Section 3.2), and the result (Supporting Information Figure S5B) shows that a plausible T_2_ map can be obtained; but again, there is little impact on *f* and $T_{1\text{D}}^{s}$ estimation.

### 5.4 Determining subspace rank

The embodiment of MT-MRF used in this work was chosen to emulate a “standard” MT measurement that cycles over different saturation settings with the same flip angle; it has the interesting property that all signal variation is MT-mediated. One important side effect of this is that parameter estimation is not possible for substances without a semisolid compartment. However, the fairly simple time structure of this specific sequence leads to smooth temporal variations such that the generated dictionaries have an effective rank of 5. Time domain signals from representative tissues could be reconstructed with < 0.75% error using the reduced basis. The presented images used rather long acquisitions (~25 minutes), which were necessary because ihMT contrast is rather weak.

In one sense, the images are highly undersampled since approximately 9 complete volumes of *k*-space data were acquired in order to reconstruct a time-variable cycle of 1200. But given the effective rank of 5, the reconstruction problem is overdetermined, although the effective degree of undersampling does not map uniformly across the singular images.

By its nature, the sequence does not probe all degrees of freedom available. This may explain the low rank and inability to measure more parameters to high precision. Nevertheless, we were able to measure some important parameters such as *f* and $T_{1\text{D}}^{s}$. Estimation accuracy is hard to assess because no “gold standard” measures exist (particularly for ihMT), but our findings are similar to those reported elsewhere. $T_{1}^{f}$ was also estimated, although analysis showed that this parameter may be more biased due a fixed $T_{2}^{f}$ that can in turn change with pathology and tissue type. In vivo $T_{1}^{f}$ values (Table 1) show significant GM–WM differences but slight underestimation compared to the literature.^39,40^ Our fixed, high $T_{1\text{Z}}^{s}$ value compared to Wang et al.^43^ may contribute to this observation.

Despite some limitations of the quantification presented here, reasonable and robust estimates are obtained for this simplest of MT-MRF implementations. Future work will consider combining our multiband pulses with more normal MRF sequences that also modulate flip angle and include other contrast preparation pulses. Modulating RF frequency offsets may allow $T_{1D}^{s}$ and $T_{2}^{S}$ dependence to be disentangled. These sequences could be optimized for precision (rather than maximum ihMT contrast) using CRLB-based methods^32^ to boost parameter estimation capabilities.

## 6 Conclusion

This work presents a cyclic-steady state sequence using multiband pulses with modulated off-resonance power. As presented, the method is a proof-of-concept that uses the machinery of modern MR fingerprinting on a sequence with constant flip angle, which should not be able to perform any parameter estimation. We show that MT-mediated signal changes are large and can be used to permit semiquantitative and limited quantitative parameter estimation. Future embodiments of this method will involve more general integration with MRF, using the multiband pulse off-resonance bands as an extra parameter to vary alongside others already used for fingerprinting.

## Supplementary Material

Supporting Information

## Figures and Tables

**Table 1:** Summary of in vivo ihMTR values and MT-MRF quantitative parameter estimates

ihMTR (%)	ss-ihMT					MT-MRF			
	CST		Frontal WM	Cortical GM		CST	Frontal WM		Cortical GM
Subject 1	4.96 ± 0.41		2.91 ± 0.41	2.06 ± 0.18		5.71 ± 0.17	4.51 ± 0.19		2.96 ± 0.35
Subject 2	4.58 ± 0.54		3.11 ± 0.50	1.82 ± 0.12		5.52 ± 0.18	4.66 ± 0.11		2.88 ± 0.24
Subject 3	4.10 ± 0.54		2.57 ± 0.37	1.71 ± 0.47		5.69 ± 0.19	4.52 ± 0.31		2.87 ± 0.23
Subject 4	4.44 ± 0.40		3.08 ± 0.37	2.51 ± 0.45		5.89 ± 0.18	4.73 ± 0.21		2.67 ± 0.37
Subject 5	4.19 ± 0.64		2.63 ± 0.54	2.12 ± 0.37		5.31 ± 0.15	4.65 ± 0.37		2.99 ± 0.34
	**f**			** $T_{1\text{D}}^{s}(\text{ms})$ **			** $T_{1}^{f}(\text{ms})$ **		
	**CST**	**Frontal WM**	**Cortical GM**	**CST**	**Frontal WM**	**Cortical GM**	**CST**	**Frontal WM**	**Cortical GM**
Subject 1	0.095 ± 0.003	0.097 ± 0.006	0.081 ± 0.004	6.22 ± 0.12	3.64 ± 0.34	2.66 ± 0.35	627 ± 12	686 ± 43	1030 ± 49
Subject 2	0.094 ± 0.001	0.094 ± 0.005	0.070 ± 0.003	5.55 ± 0.14	3.64 ± 0.41	2.64 ± 0.23	612 ± 11	678 ± 29	926 ± 57
Subject 3	0.093 ± 0.002	0.095 ± 0.004	0.075 ± 0.003	5.94 ± 0.13	3.76 ± 0.26	2.73 ± 0.25	592 ± 10	690 ± 42	1000 ± 69
Subject 4	0.099 ± 0.002	0.091 ± 0.003	0.076 ± 0.003	5.48 ± 0.13	3.71 ± 0.22	2.64 ± 0.28	622 ± 12	698 ± 23	1100 ± 48
Subject 5	0.092 ± 0.001	0.089 ± 0.005	0.074 ± 0.004	5.24 ± 0.17	3.75 ± 0.25	2.64 ± 0.30	605 ± 29	688 ± 31	1040 ± 39

*Notes:* Top table shows ihMTR values obtained from different regions-of-interest: frontal WM, cortical GM, and CST in the axial slices from 5 subjects (1-5; left-to-right) in Figure 6. Bottom table shows MT-MRF quantitative parameter estimates obtained for the same axial regions-of-interest from the 5 subjects in Figure 7. Abbreviations: CST, corticospinal tract; GM, gray matter; ihMTR, inhomogeneous magnetization transfer ratio; MT-MRF, magnetization transfer-mediated MR fingerprinting; ss-ihMT, steady-state inhomogeneous magnetization transfer; WM, white matter.

**Table 2:** Comparison of phantom dictionary fitting results for MT-MRF and ss-ihMT

	$T_{2}^{f}=84\text{ms}$		$T_{2}^{f}=130\text{ms}$	
	MT-MRF	ss-ihMT	MT-MRF	ss-ihMT
BSA				
$T_{1}^{f}(\text{s})$	1.35 ± 0.08	1.43 ± 0.30	1.05 ± 0.04	1.83 ± 0.38
*f*	0.081 ± 0.005	0.087 ± 0.009	0.082 ± 0.005	0.075 ± 0.009
$T_{1\text{D}}^{s}(\text{ms})$	0.44 ± 0.38	1.3 ± 3.0	0.42 ± 0.39	2.7 ± 3.6
PL161				
$T_{1}^{f}(\text{s})$	2.02 ± 0.15	1.12 ± 0.15	1.40 ± 0.10	1.41 ± 0.18
*f*	0.177 ± 0.008	0.188 ± 0.014	0.175 ± 0.006	0.168 ± 0.014
$T_{1\text{D}}^{s}(\text{ms})$	24.6 ± 0.9	26 ± 4.0	24.5 ± 1.0	28.4 ± 4.1

*Notes*: Two independent fits were performed - one with free pool T_2_ more aligned to BSA (84 ms) and another with free pool T_2_ more aligned to PL161 (130 ms). Abbreviations: BSA, bovine serum albumin; PL161, prolipid 161.

## Data Availability

According to UK research councils' Common Principles on Data Policy and Wellcome Trust's Policy on data, software and materials management and sharing, all code used to generate the simulation results in this study can be found at: https://github.com/mriphysics/MT-MRF (hash 8b8089a was used for the presented results). This excludes in vivo data because of the terms of the ethical approval under which they were acquired.
