# Supplementary material for: An MR fingerprinting approach for quantitative inhomogeneous magnetization transfer imaging
Source: Magn Reson Med. Author manuscript; Available in PMC 2023 Jan 3. (PMC7614010; doi:10.1002/mrm.28984)
Supplement: Supporting Information [file EMS157953-supplement-Supporting_Information.pdf]

Supporting Information 1: Steady-State Signal Calculation

The time-evolution of magnetization can first be written as Eq. S1 and then reformulated as Eq. S2, where $\mathbf{M} = \left[M_x^f\ M_y^f\ M_z^f\ M_Z^{s1} M_Z^{s2}\ M_D^{s2}\right]^T$.

$$\dot{\mathbf{M}} = \mathbf{AM} + \mathbf{b} \qquad [S1]$$

$$\dot{\widetilde{\mathbf{M}}} = \widetilde{\mathbf{A}}\widetilde{\mathbf{M}} \qquad [S2a]$$

$$\widetilde{\mathbf{M}} = \begin{bmatrix} \mathbf{M} \\ 1 \end{bmatrix} \qquad [S2b]$$

$$\widetilde{\mathbf{A}} = \begin{bmatrix} \mathbf{A} & \mathbf{b} \\ \mathbf{0} & \end{bmatrix} \qquad [S2c]$$

Here, 1 is a scalar value and $\mathbf{0}$ represents a row vector matching the dimensionality of $\mathbf{A}$ and $\mathbf{b}$. The resultant equation is homogeneous and the solution over time increment TR (during which $\widetilde{\mathbf{A}}$ is constant) is: $\widetilde{\mathbf{M}}(t + \mathrm{TR}) = \widetilde{\mathbf{A}}(t)\widetilde{\mathbf{M}}(t)$. For MT-MRF, we alternate between RF pulses with one, two and three bands (1B/2B/3B). For a given RF pulse, the effect on the magnetization is:

$$\mathbf{R} = \exp(\langle \mathbf{\Omega} \rangle \tau) \qquad [S3]$$

where $\mathbf{\Omega}$ is defined fully by Equation 4 in Malik *et al.* (1) and the angle brackets indicate a time average is taken over the RF pulse of duration τ. Note that here, 'exp' is a matrix exponential. After each pulse, we must apply the operation $\mathbf{S}$ to account for relaxation and exchange processes:

$$\mathbf{S} = \exp\left(\begin{bmatrix} \mathbf{\Lambda} & \mathbf{C} \\ \mathbf{0} & \end{bmatrix} \mathrm{TR}\right) \qquad [S4]$$

where again, $\mathbf{\Lambda}$ and $\mathbf{C}$ are defined in Malik *et al.* (1) (Equation 2). Lastly, we can apply the operation $\mathbf{D} = \mathrm{diag}[-1\ -1\ 1\ 1\ 1\ 1\ 1]$ to account for bSSFP phase alternation.

The MT-MRF sequence consists of a repeating cycle of n_{MB} 2B pulses, $n_{1\mathrm{B}}$ 1B pulses, n_{MB} 3B pulses, and $n_{1\mathrm{B}}$ 1B pulses such that the total cycle duration is $\mathrm{N_{cycle}} = 2(n_{1\mathrm{B}} + n_{\mathrm{MB}})$. Taking the beginning of the 2B period as the reference point, steady-state magnetization can be calculated by enforcing the periodic boundary condition:

$$\widetilde{\mathbf{M}}(t + \mathrm{N_{cycle}TR}) = (\mathbf{R_{1B}DS})^{n_{1\mathrm{B}}}(\mathbf{R_{3B}DS})^{n_{3\mathrm{B}}}(\mathbf{R_{1B}DS})^{n_{1\mathrm{B}}}(\mathbf{R_{2B}DS})^{n_{2\mathrm{B}}}\,\widetilde{\mathbf{M}}(t) \qquad [S5]$$

where $\mathbf{R_{1B}}$ corresponds to 1B pulses, etc. This equation can be solved by identifying the eigenvector of matrix $(\mathbf{R_{1B}DS})^{n_{1\mathrm{B}}}(\mathbf{R_{3B}DS})^{n_{3\mathrm{B}}}(\mathbf{R_{1B}DS})^{n_{1\mathrm{B}}}(\mathbf{R_{2B}DS})^{n_{2\mathrm{B}}}$ with eigenvalue 1, as described in (1).

Supporting Information 2: Multiband Pulse Corrections

MT-MRF switches between single-band and multiband pulses with the aim that on-resonance components of these pulses are invariant. When conducting non-phase-encoded experiments, we noticed that significant and unexpected signal fluctuations occurred at the point where pulse type changed and found this was caused by the on-resonance lobes having a slightly different amplitude and phase. A pick-up coil measurement utility already implemented on the scanner was used to characterize these discrepancies for each scan. The origin could not be found but was suspected to be from the RF hardware. Errors were not easily predictable from scan parameters but were found to be consistent as long as scan parameters were unchanged.

Example measurements are plotted in Figure S1 that displays the measured RF pulses in the frequency domain. When zooming in on the on-resonance band, it is clear that there are some discrepancies between the pulses. Complex scaling factors were calculated to make the behavior of each multiband pulse (i.e. 2B and 3B) match that of the single-band (1B) pulse at $\Delta = 0$. The MT-MRF pulse sequence was implemented to allow user-defined values to be entered for these scaling factors. Table S1 shows examples of the required scaling factors.

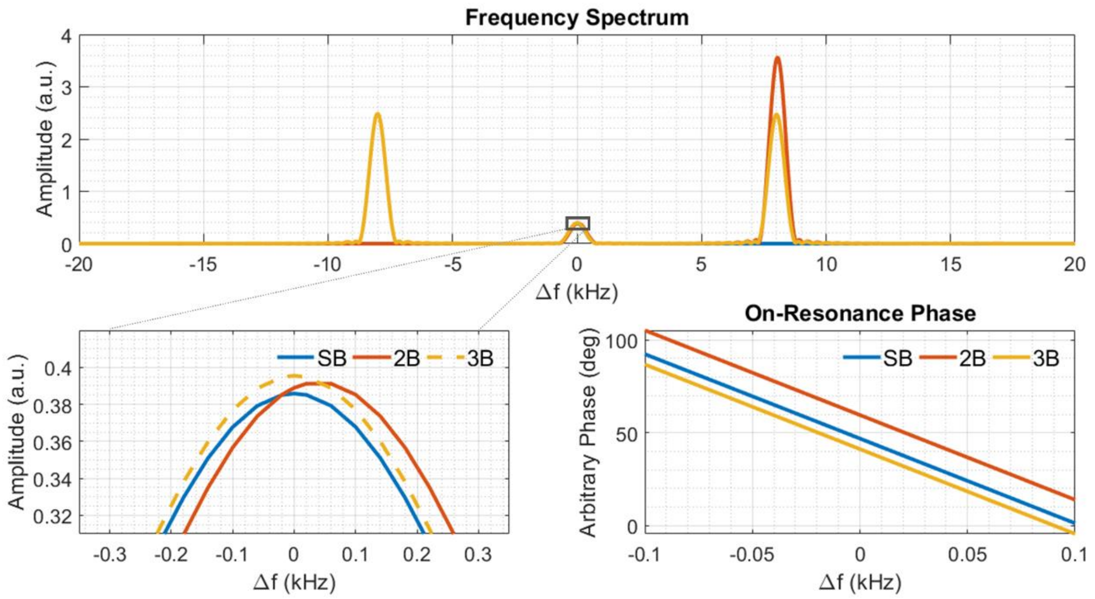

Figure S1: Plots used to derive amplitude and phase correction factors between single-band (SB), 2-band (2B) and 3-band (3B) pulses. Note how the on-resonance lobes and phases of each pulse are slightly offset from one another. These offsets are overcome using the example factors shown in Table S1.

Table S1: Summary of multiband scaling factors used during the phantom and *in vivo* experiments.

**2B Phase Factor (˚)**	**3B Phase Factor (˚)**	**2B Amplitude Factor**	**3B Amplitude Factor**
-12.7	5.7	1.008	1.025

Non-phase-encoded phantom scans were then repeated to ensure that fluctuations were removed, and this is confirmed in Figure S2 (below).

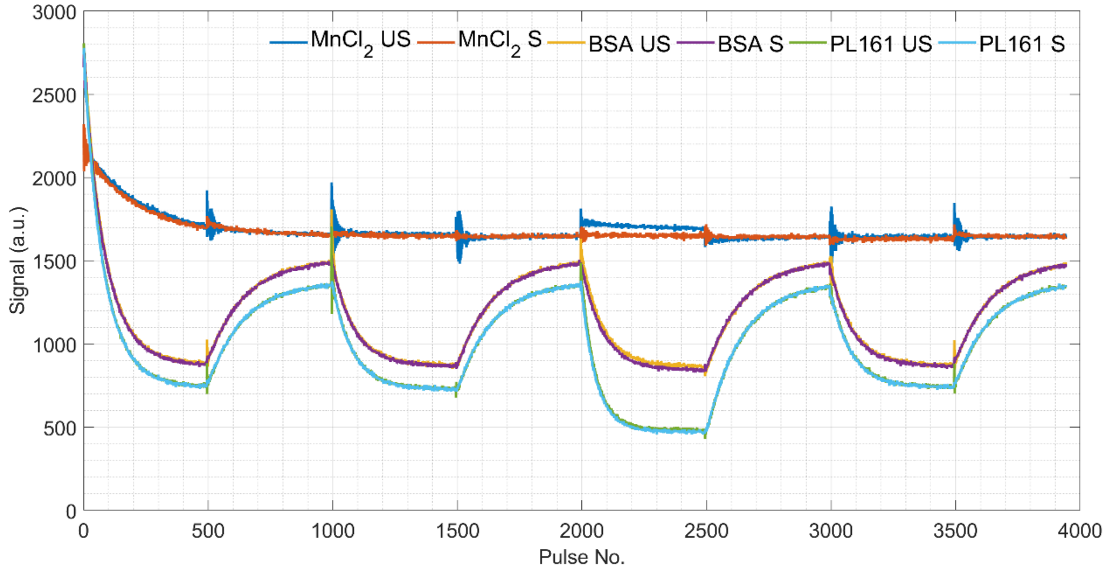

Figure S2: Example 1D signal profiles from each phantom before (unscaled, US) and after (scaled, S) amplitude and phase correction. Large fluctuations at transitions between pulse types are significantly reduced and removed for BSA and PL161. The pulse cycle used to obtain these plots is: {500 $2B^+$, 500 SB, 500 $2B^-$, 500 SB, 500 3B, 500 SB etc.} so does not match the actual acquisition scheme used in the manuscript; it was used for debugging purposes only. $2B^+$ refers to a 2-band pulse with a positive off-resonance lobe and $2B^-$ has an equal but opposite negative off-resonance lobe. Though the latter is not used in our phantom and *in vivo* experiments, it can be used to generate ihMT contrast when combined with a matched $2B^+$ pulse to approximate dual frequency off-resonance.

Supporting Information 3: Supplementary Phantom Results

To supplement Table 2 in the manuscript, below are single-slice maps resulting from the two separate dictionary fits to MT-MRF phantom data. Note the similarity between MT parameter estimates when either T_2^f = 84ms or T_2^f = 130ms. Only T_1^f values change slightly due to an expected T_2^f dependence.

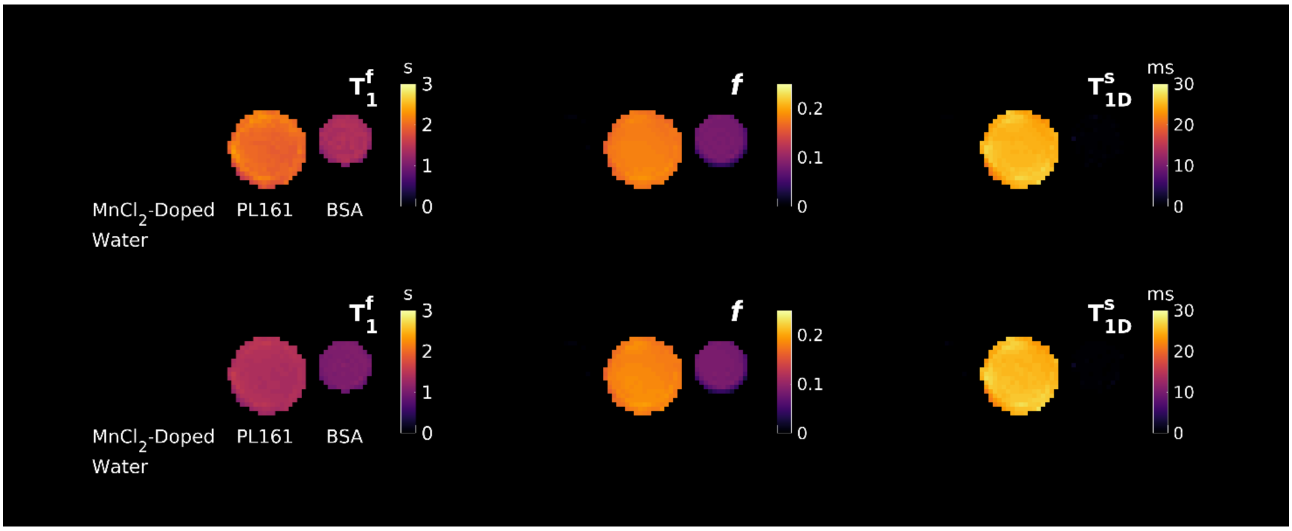

Figure S3: *Top*: MT-MRF parameter maps obtained using a GM-WM average parameter set with T_2^f = 84ms (Section 3.2). *Bottom*: Equivalent parameter maps but assuming a more 'PL161-like' fixed value for T_2^f (130ms) only.

Supporting Information 4: Supplementary *in vivo* Results

To supplement the inter-subject ihMTR comparison shown in Table 1 of the manuscript, below is an equivalent comparison of corresponding MTR values between MT-MRF and ss-ihMT.

Table S2: MTR values obtained from different regions-of-interest for the axial slices of the five subjects in Figure 6.

**MTR (%)**	**ss-ihMT**			**MT-MRF**		
	**CST**	**Frontal WM**	**Cortical GM**	**CST**	**Frontal WM**	**Cortical GM**
**Subject 1**	47.4 ± 1.8	46.6 ± 0.8	42.5 ± 1.9	36.7 ± 0.7	38.4 ± 0.9	32.0 ± 2.3
**Subject 2**	47.4 ± 1.5	46.6 ± 0.7	44.7 ± 0.6	36.1 ± 0.5	37.1 ± 0.8	33.4 ± 0.9
**Subject 3**	46.1 ± 1.9	45.6 ± 0.9	42.3 ± 1.6	35.3 ± 0.9	37.5 ± 0.9	31.8 ± 1.0
**Subject 4**	47.6 ± 0.8	46.8 ± 0.9	42.3 ± 0.8	36.1 ± 0.4	37.0 ± 0.9	31.7 ± 1.4
**Subject 5**	46.6 ± 0.9	46.1 ± 1.1	42.4 ± 1.7	37.1 ± 1.5	37.2 ± 1.1	31.8 ± 1.7

For Subject 2 (male, aged 25), two identical MT-MRF scans were completed approximately eight months apart. Figure S4 presents a central axial slice from each dataset following registration. Table S3 compares measurements in corresponding matched regions-of-interest.

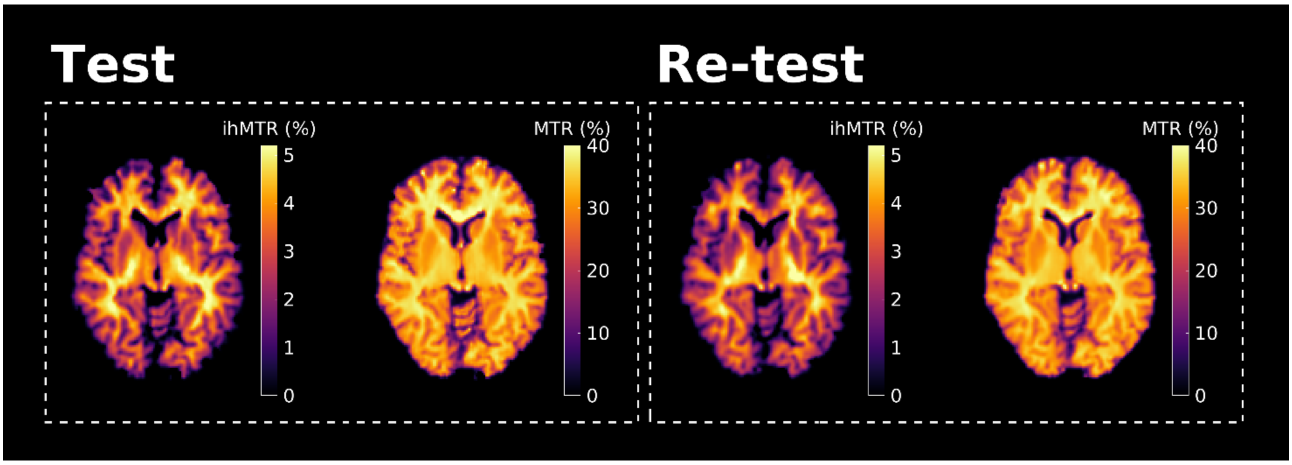

Figure S4: Equivalent central axial slices from the same healthy subject but two separate acquisitions. ihMTR and MTR contrasts from MT-MRF are repeatable according to the region-of-interest measurements shown in Table S3.

Table S3: ihMTR and MTR values from three different brain regions in the axial slices shown in Figure S4.

	**ihMTR (%)**			**MTR (%)**		
	**CST**	**Frontal WM**	**Cortical GM**	**CST**	**Frontal WM**	**Cortical GM**
**Test**	5.02 ± 0.21	4.72 ± 0.29	3.06 ± 0.25	35.4 ± 0.7	37.9 ± 0.9	31.8 ± 1.5
**Re-test**	4.98 ± 0.51	4.75 ± 0.13	2.95 ± 0.27	35.8 ± 0.7	37.3 ± 1.0	31.9 ± 1.1

Supporting Information 5: Additional *in vivo* Dictionary Fitting Results

Fixing T^s_{1Z} to a Lower Value

Recently, Wang *et al*. suggested that T^s_{1Z} is much lower than is usually assumed in MT literature. (2) Therefore, we repeat dictionary fits for Subject 1 using a lower, brain-average T^s_{1Z} = 200ms.

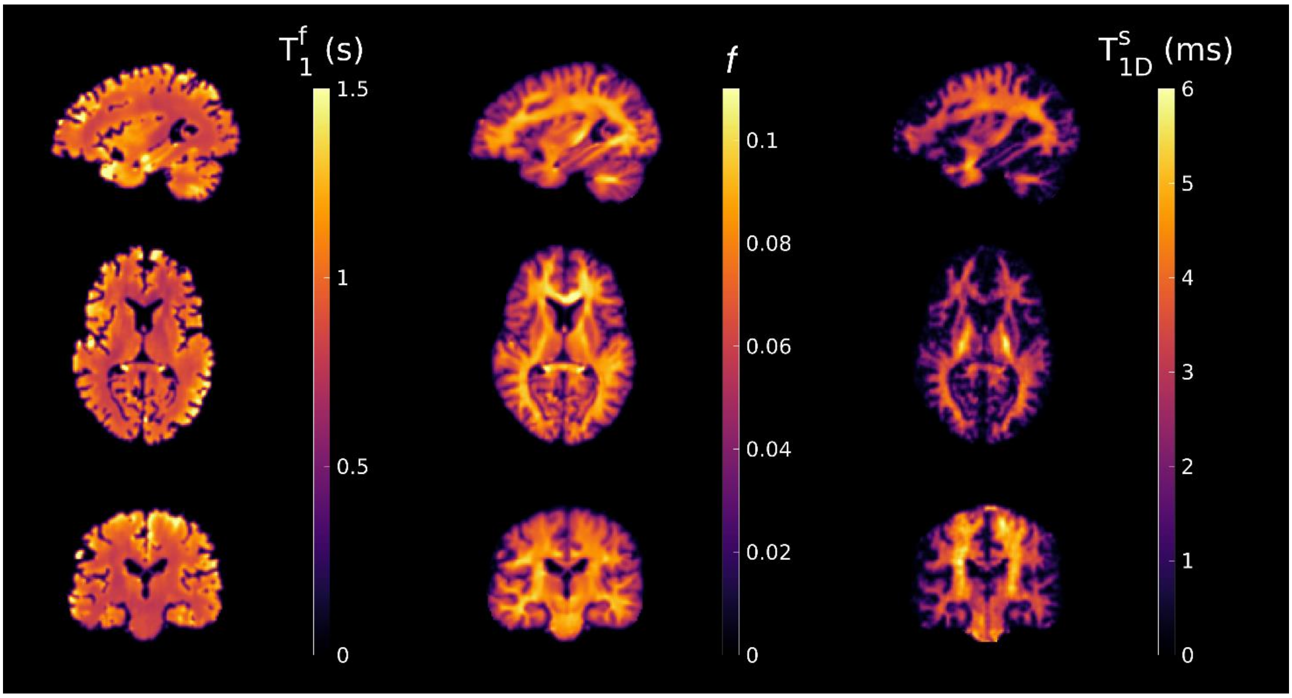

Figure S5a: Dictionary fitting results for Subject 1 assuming lower T^s_{1Z}. Compared to Figure 8, T^f_1 estimates increase since free and semisolid T_1 are coupled to one another, whilst estimates for f and T^s_{1D} are mostly unchanged.

Fitting for T_2^f

From Figure 3, Combination 4 seems to also give reasonable estimation precision and so dictionary fits are repeated for Subject 1 but for the case where T_2^f is estimated and T_1^f is fixed at 1.36s. (3)

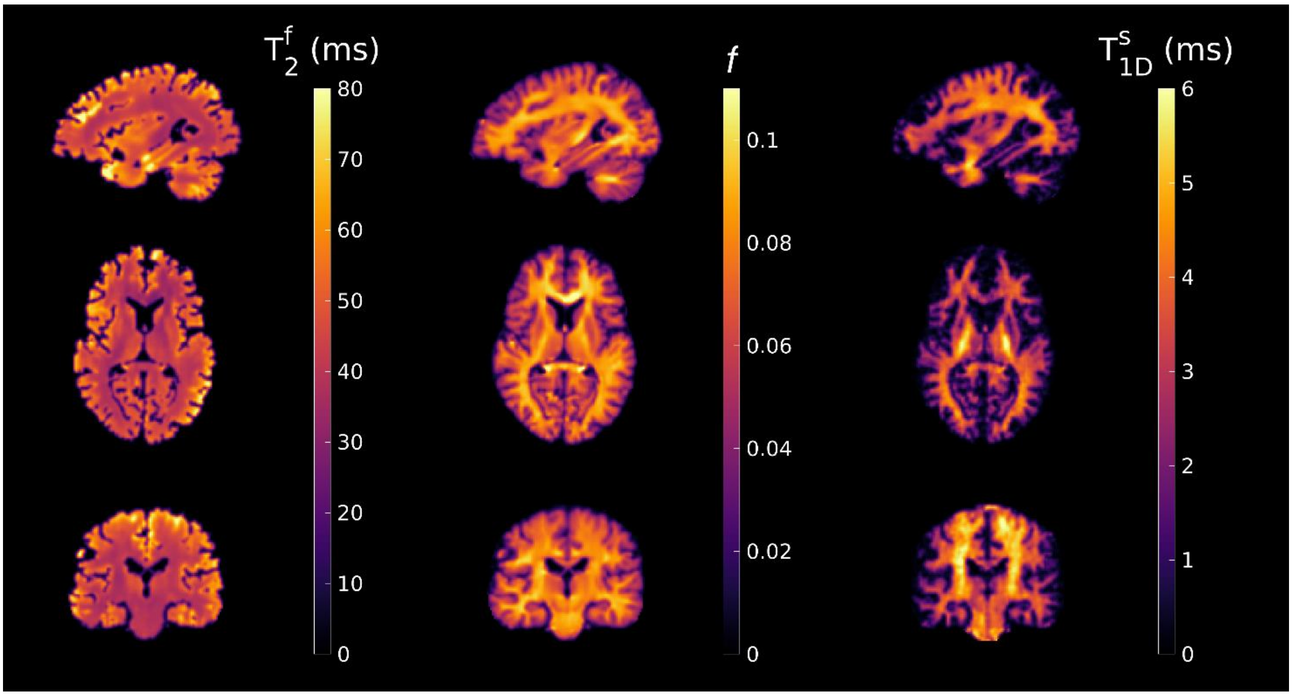

Figure S5b: Dictionary fitting results for Subject 1 assuming fixed T_1^f but estimating T_2^f. T_2^f maps show similar contrast to those for T_1^f but once more, estimates for f and T_{1D}^s are almost indiscernible from those reported in Figure 8.

Supporting Information 6: Investigation of Bias in Dictionary Fits

To investigate potential biases in the dictionary matching process, fitting was performed using a series of numerical phantoms. A range of estimated parameters (T_1^f, f and T_{1D}^s) were fitted, while the fixed parameters (T_{1Z}^s, T_2^f, T_2^s and k) were changed. In each case, a time-domain signal from the relevant parameter combination was forward simulated, Gaussian noise was added (SNR = 40 defined with respect to the signal mean over time), and the resultant signal transformed to a low-rank basis by $\mathbf{U_R}$. The parameters were then estimated via dictionary matching and 100 noise realizations were used for each parameter combination. Figure S6a shows estimated parameter values as f and T_{1D}^s are changed and the 'hidden' parameters (T_{1Z}^s, T_2^f, T_2^s and k) were given the same values as those used to create $\widetilde{\mathbf{D}}$.

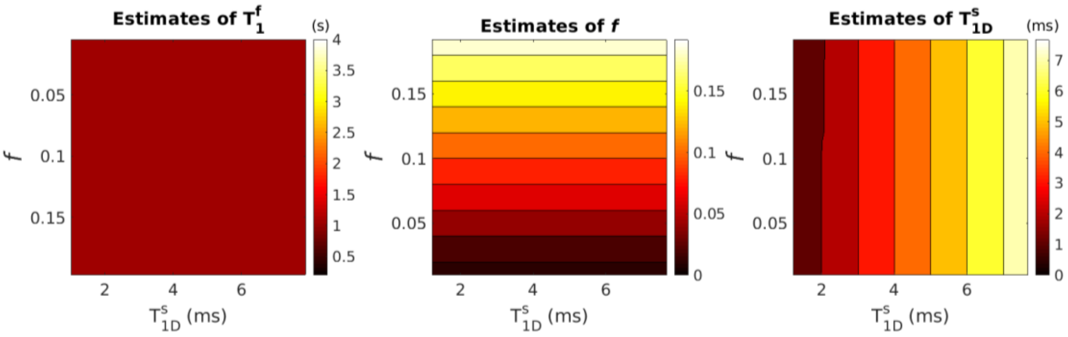

Figure S6a: Mean estimates of T_1^f, f and T_{1D}^s over 100 noise realizations as ground-truth values of f and T_{1D}^s are changed, T_1^f = 1.09s and all remaining parameters are fixed to those used to generate $\widetilde{\mathbf{D}}$. T_1^f is accurately estimated, showing a constant value. For the middle and right-most subplots, contours represent lines of constant estimated parameter values and so both f and T_{1D}^s are also well estimated. Slight deviation of T_{1D}^s is seen for the combination of high f and low T_{1D}^s.

Figure S6a shows that when the fixed parameters match their values used to generate the dictionary, quantification is very accurate. To test the robustness as 'hidden' fixed parameters change, the above process was repeated while incrementing these parameters over inclusive ranges (T_{1Z}^s = 0.6-1.3s, T_2^f = 50-120ms, T_2^s = 6-13µs and k = 45-80s^{-1}) while still estimating T_1^f, f and T_{1D}^s using $\widetilde{\mathbf{D}}$. Figure S6b contains animated plots that cycle through ranges of each 'hidden' parameter and the subsequent violin plots (4) summarize the error in these estimates. Generally, f and T_{1D}^s estimates are stable as T_{1Z}^s and T_2^f values are changed (contours in Figure S6b remain horizontal and vertical respectively), whilst T_1^f estimation mainly depends on the latter (note the changing shade of red in the corresponding subplot). Due to the influence of T_2^s on generating the super-Lorentzian lineshape, T_{1D}^s estimates change significantly with T_2^s. Comparatively smaller dependencies exist with respect to k, though f is somewhat less accurately estimated towards the lower and upper bounds of the investigated range.

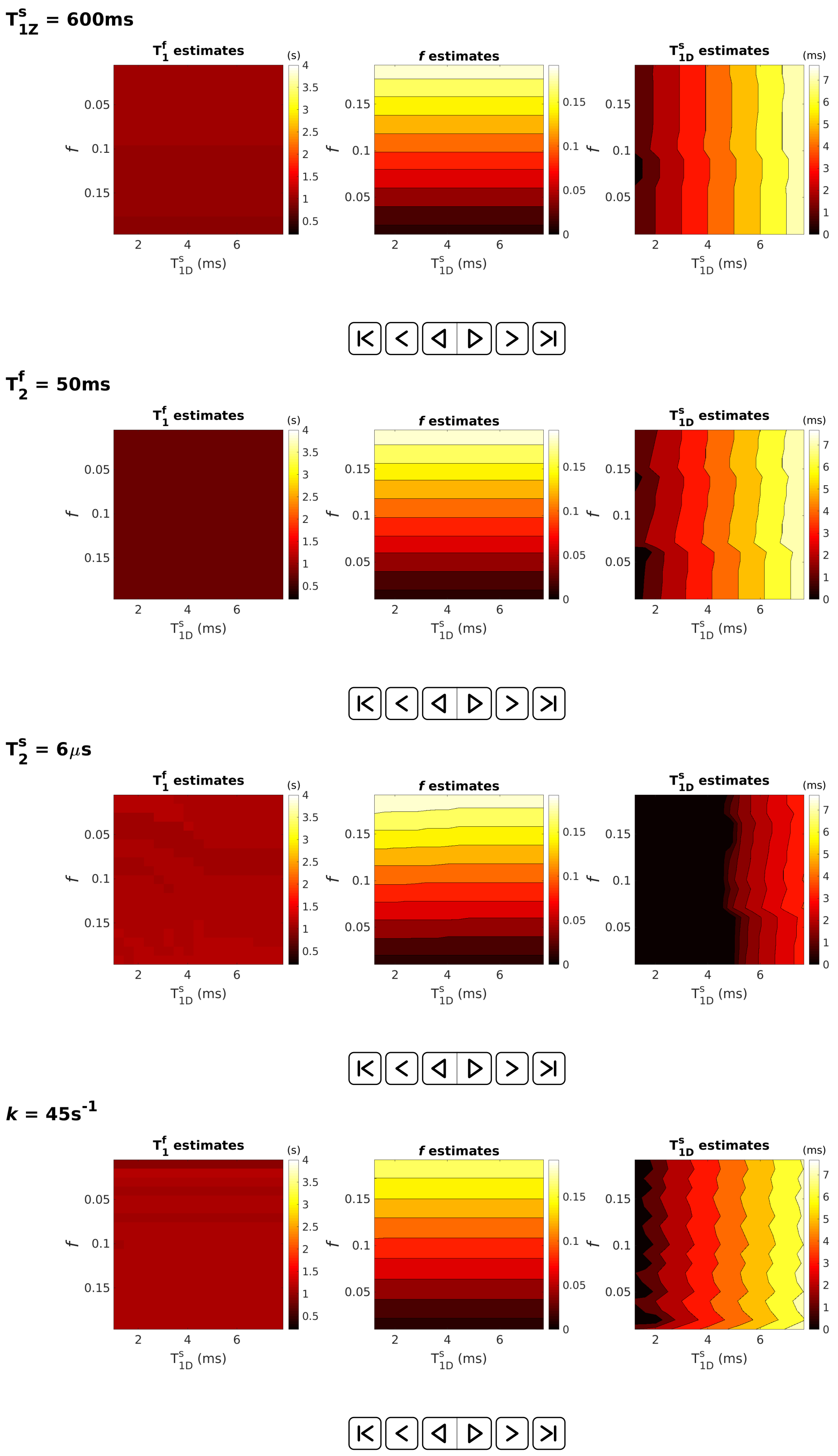

Figure S6b: Heat maps produced as in Figure S6a but with *hidden* parameter values changed according to the titles above each of the four subplots. To navigate through different maps this document needs to be open on a JavaScript-supporting PDF viewer, such as Adobe Acrobat Reader.

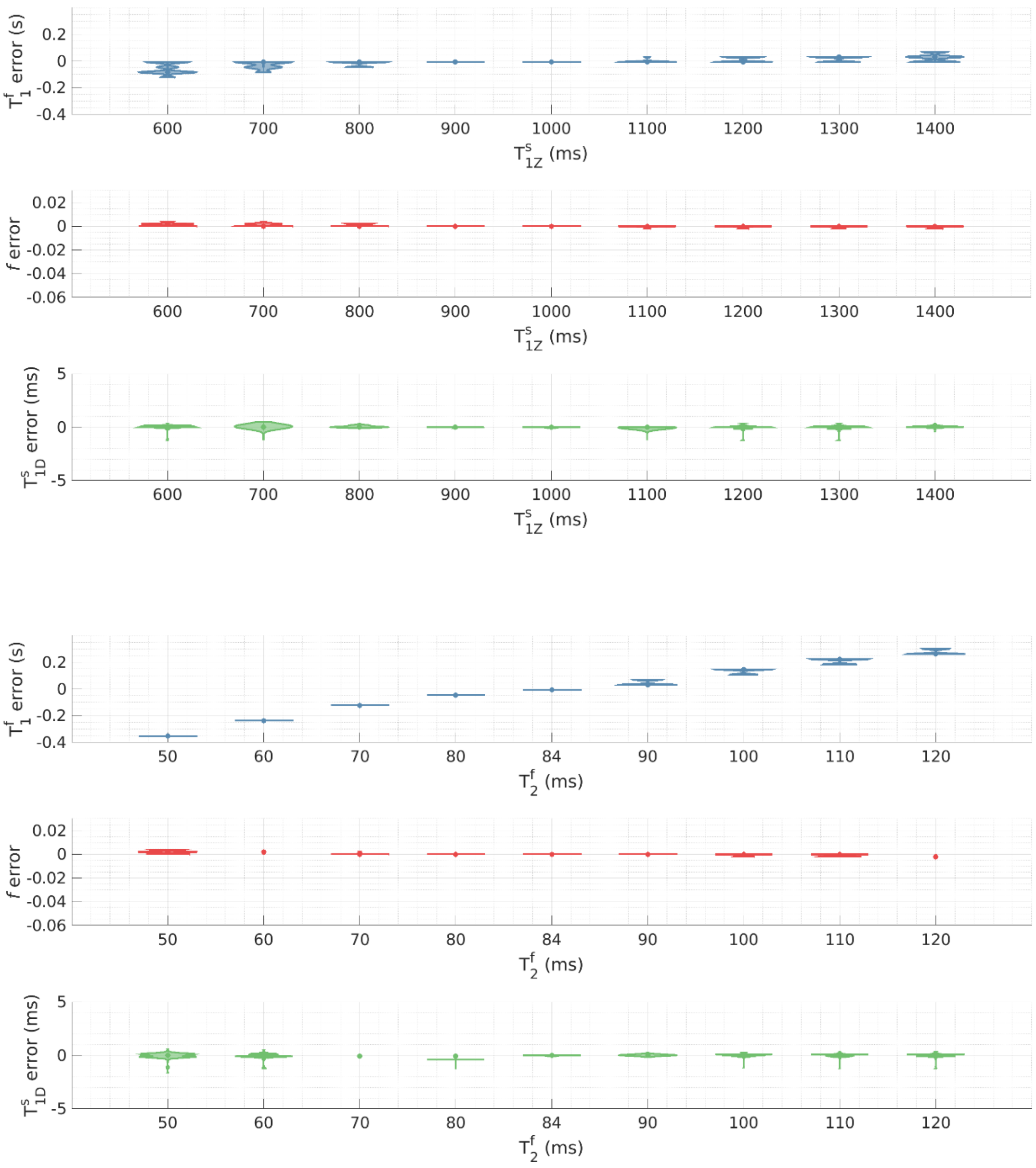

Figure S6c: Bias in parameter estimates as T_{1Z}^{s} and T_2^f values are changed. T_1^f is highly correlated with T_2^f but estimates of f and T_{1D}^{s} are robust even at the lower and upper bounds of the investigated ranges.

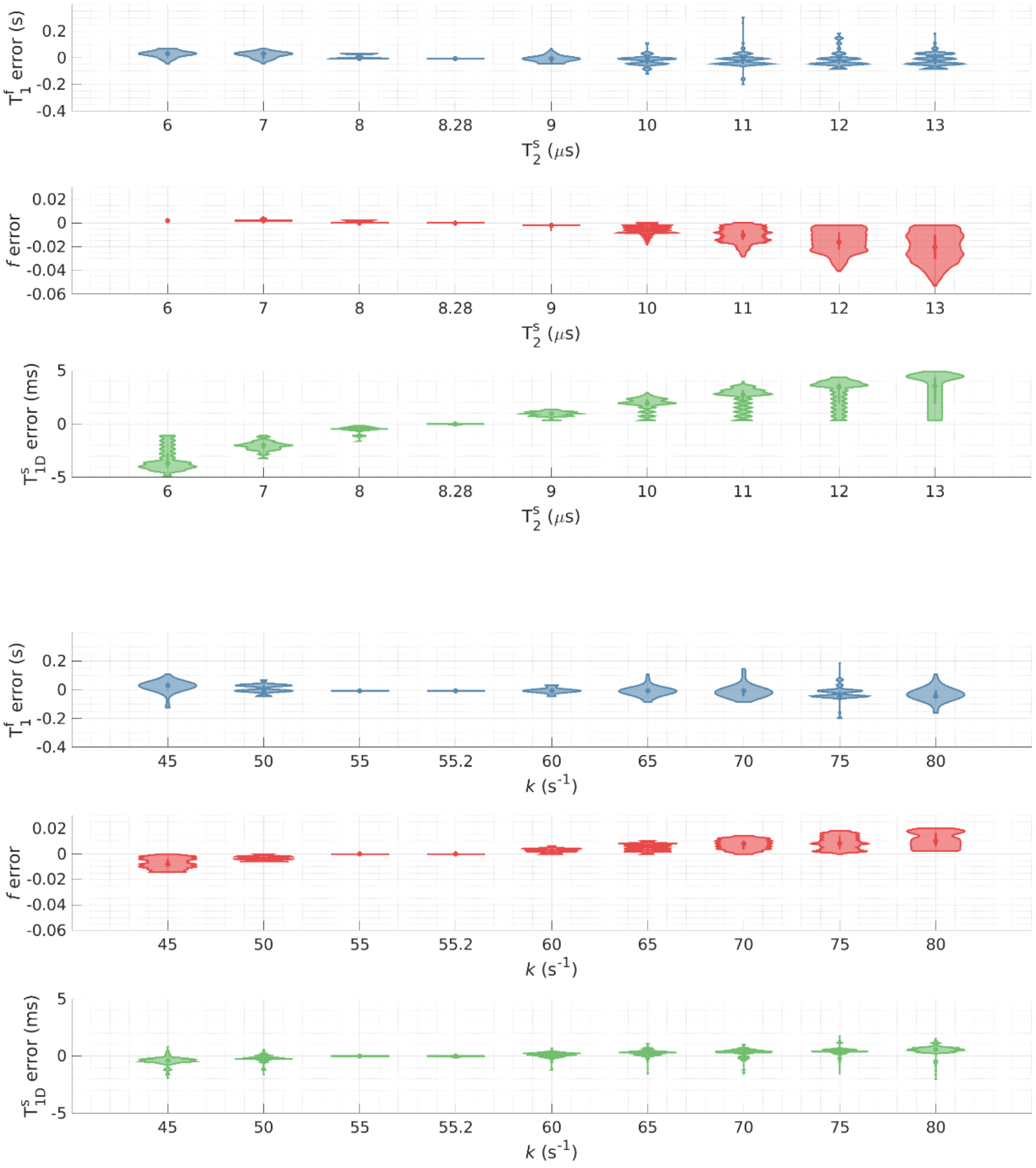

Figure S6d: Bias in parameter estimates as T_2^s and k values are changed. Whereas T_{1D}^s bias is consistent as k is incremented, inaccuracies in f become apparent further from the ground-truth value of 55.2s^{-1}. Much larger biases occur when modifying the fixed value of T_2^s since this parameter is used to describe the super-Lorentzian lineshape.

Dictionary fitting was also performed to *in vivo* data by incrementing our previously assumed fixed parameter values (in $\widetilde{\mathbf{D}}$) by ±10%. Difference maps were computed between these two to reveal possible parameter dependencies, corroborating the sources of bias identified from simulations above.

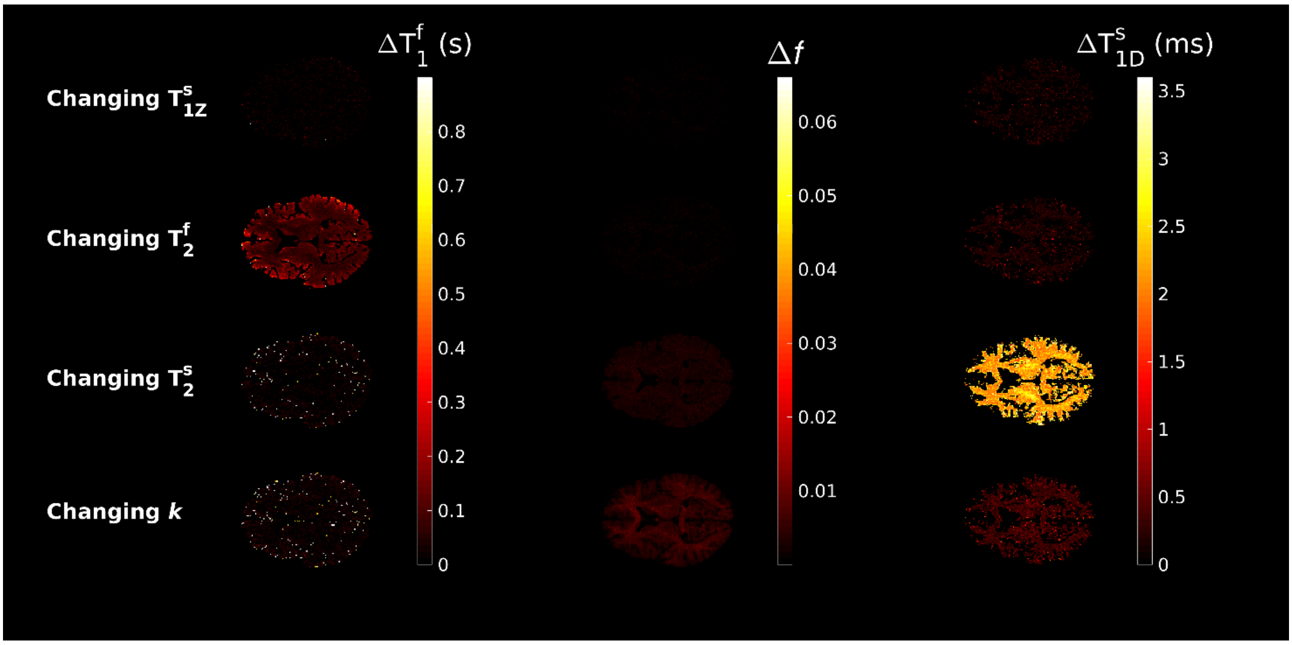

Figure S6e: Difference maps for each estimated parameter given a ±10% change in the fixed parameters stated on the left-hand side. Each estimated parameter seems invariant to small changes in T_{1Z}^s; T_1^f is most coupled to T_2^f; T_{1D}^s shows considerable dependence on T_2^s; and f is slightly influenced by k. Colorbars for each estimated parameter change (ΔX, where X is the estimated parameter) are chosen to cover 60% of the ranges used for these quantities in other figures.

Supporting Information References

1. Malik SJ, Teixeira RPAG, West DJ, Wood TC, Hajnal JV. Steady-state imaging with inhomogeneous magnetization transfer contrast using multiband radiofrequency pulses. Magn. Reson. Med. 2020;83:935–949.
2. Wang Y, van Gelderen P, de Zwart JA, Duyn JH. B0-field dependence of MRI T1 relaxation in human brain. Neuroimage 2020;213:1–11.
3. Varma G, Girard OM, Mchinda S, et al. Low duty-cycle pulsed irradiation reduces magnetization transfer and increases the inhomogeneous magnetization transfer effect. J. Magn. Reson. 2018;296:60–71.
4. Bechtold, Bastian, 2016. Violin Plots for MATLAB, GitHub Project https://github.com/bastibe/Violinplot-Matlab, DOI: 10.5281/zenodo.4559847
